# Comparison of Deep Transfer Learning Against Contrastive Learning in Industrial Quality Applications for Heavily Unbalanced Data Scenarios When Data Augmentation Is Limited

**DOI:** 10.3390/s25103048

**Published:** 2025-05-12

**Authors:** Amir Farmanesh, Raúl G. Sanchis, Joaquín Ordieres-Meré

**Affiliations:** Department of Organization Engineering, Business Administration and Statistics, Universidad Politécnica de Madrid, 28040 Madrid, Spain; amir.farmanesh@upm.es (A.F.); raul.g.sanchis@upm.es (R.G.S.)

**Keywords:** quality inspection, imbalanced data, deep transfer learning, contrastive learning, industrial vision, limited data augmentation, industrial defect detection

## Abstract

AI-oriented quality inspection in manufacturing often faces highly imbalanced data, as defective products are rare, and there are limited possibilities for data augmentation. This paper presents a systematic comparison between Deep Transfer Learning (DTL) and Contrastive Learning (CL) under such challenging conditions, addressing a critical gap in the industrial machine learning literature. We focus on a galvanized steel coil quality classification task with acceptable vs. defective classes, where the vast majority of samples (>95%) are acceptable. We implement a DTL approach using strategically fine-tuned YOLOv8 models pre-trained on large-scale datasets, and a CL approach using a Siamese network with multi-reference design to learn robust similarity metrics for one-shot classification. Experiments employ k-fold cross-validation and a held-out gold-standard test set of coil images, with statistical validation through bootstrap resampling. Results demonstrate that DTL significantly outperforms CL, achieving higher overall accuracy (81.7% vs. 61.6%), F1-score (79.2% vs. 62.1%), and precision (91.3% vs. 61.0%) on the challenging test set. Computational analysis reveals that DTL requires 40% less training time and 25% fewer parameters while maintaining superior generalization capabilities. We provide concrete guidance on when to select DTL over CL based on dataset characteristics, demonstrating that DTL is particularly advantageous when data augmentation is constrained by domain-specific spatial patterns. Additionally, we introduce a novel adaptive inspection framework that integrates human-in-the-loop feedback with domain adaptation techniques for continuous model improvement in production environments. Our comprehensive comparative analysis offers empirically validated insights into performance trade-offs between these approaches under extreme class imbalance, providing valuable direction for practitioners implementing industrial quality inspection systems with limited, skewed datasets.

## 1. Introduction

Modern manufacturing demands stringent quality control, especially in industries like the automotive and steel production industries [1,2]. Visual inspection systems must rapidly detect defects or deviations to ensure product reliability and safety. Traditionally, quality inspection has relied on rule-based machine vision or manual checks, which are labor-intensive, prone to errors, and difficult to scale [3]. However, deploying deep learning in industrial quality inspection faces two critical challenges: extreme data imbalance and inherent data scarcity [4].

In real-world manufacturing environments, defective samples lie typically between 10% and 15% of all products [5], leading to severely skewed datasets that can bias model learning toward the majority class [6,7]. Additionally, collecting or augmenting data for all possible defect variations is exceptionally difficult, as standard augmentation techniques often fail to capture the full diversity of defects while maintaining their distinctive characteristics [8,9]. Unlike natural image datasets where rotations and flips preserve semantic meaning, industrial defect patterns often have spatial dependencies that must be preserved during augmentation [10,11].

**Figure 1:**
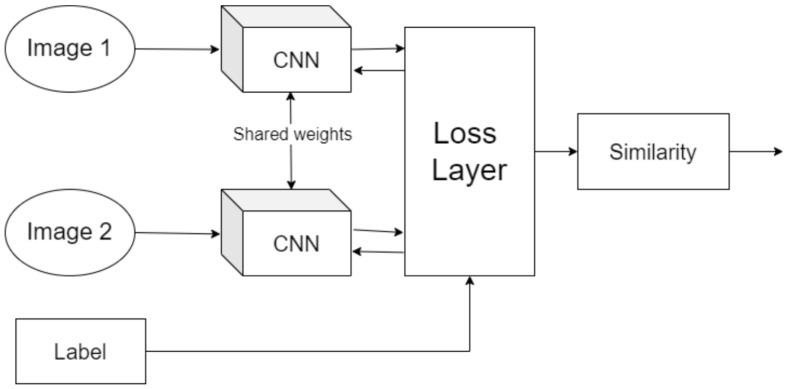
Siamese network architecture with shared CNN weights and similarity loss.

Contrastive Learning, including Siamese networks and self-supervised methods, has emerged as a promising approach for limited data scenarios [12,13,14]. CL learns an embedding space where similar samples cluster together while dissimilar samples remain distant [6,15]. This approach can capitalize on unlabeled or pairwise data to learn robust representations, potentially mitigating label imbalance by focusing on relative comparisons rather than absolute classifications [6,15,16,17]. Siamese neural networks, in particular, have demonstrated success in one-shot learning scenarios, requiring only a few examples to distinguish new classes by comparing input pairs [15,18,19,20]. In industrial contexts, Siamese models have been effectively used to verify if a part is defective by comparing it to known good references [21,22]. Recent studies have shown that CL approaches can improve minority-class accuracy [6]; for instance, Marrakchi et al. (2021) [23] demonstrated that incorporating contrastive loss helped classifiers pay more attention to underrepresented classes in medical images. Furthermore, specialized contrastive losses have been developed specifically to handle class imbalance [24,25]. Vito and Stefanus (2022) [6] proposed an Asymmetric Contrastive Loss (ACL) that assigns higher weight to minority class pairs, yielding better balanced accuracy on imbalanced binary datasets [6]. These advances suggest that CL could learn discriminative features from limited data, making it theoretically attractive for quality inspection scenarios where defects are scarce. Figure 1 visually demonstrates the Siamese architecture commonly used in Contrastive Learning, emphasizing its capability to distinguish between similar and dissimilar samples.

**Figure 2:**
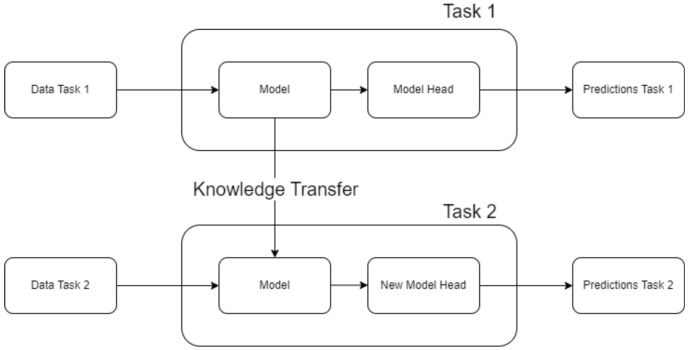
Transfer learning framework with knowledge transfer between tasks.

In parallel, Deep Transfer Learning (DTL) has emerged as a powerful alternative approach that leverages pre-trained models and fine-tunes them on target tasks [24,25,26]. By transferring generic visual features learned from large, diverse datasets, DTL can significantly boost performance on small, specialized industrial datasets [3]. Recent studies have demonstrated impressive results with this approach. For example, Lin et al. (2025) [26] achieved 98.7% accuracy in tool assembly defect detection by fine-tuning an AlexNet model, vastly outperforming networks trained from scratch on the same limited data. Similarly, Yang et al. (2023) [27] applied pre-trained CNNs (VGG16/19) combined with ensemble techniques to reliably detect 3D printing defects, noting that transfer learning reduced training data requirements by up to 60% while improving accuracy by 15% compared to non-transfer approaches. These successes indicate that DTL can effectively address data scarcity in quality inspection applications, but its comparative effectiveness against CL [23] under extreme class imbalance remains underexplored [28]. Figure 2 clearly illustrates the core principle of transfer learning, highlighting how knowledge is transferred from a pre-trained model to a target task.

Given these two promising paradigms, a critical question emerges: can Deep Transfer Learning compete with or even surpass Contrastive Learning for imbalanced industrial quality inspection tasks? While CL has gained significant attention as a solution for data-sparse problems [23], it remains uncertain whether it can outperform the conceptually simpler strategy of fine-tuning a pre-trained network, especially in real-world industrial settings with extremely skewed class distributions and domain-constrained data augmentation possibilities. This knowledge gap has significant practical implications for industrial AI implementation, as choosing the optimal approach can substantially impact inspection accuracy, implementation costs, and long-term system maintenance [29].

**Figure 3:**
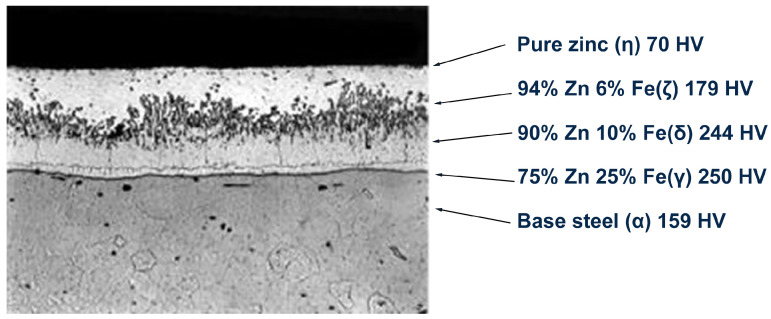
Microhardness (HV) of zinc-coated steel layers and base steel.

To address this gap, we present a comprehensive comparative analysis of DTL and CL approaches applied to an automotive steel coil coating inspection task. This application represents a quintessential industrial quality control challenge with real-world constraints. In this task, zinc-coated steel coil data are recorded by 18 sensors alongside the coils and classified by experts as “acceptable” (OK) or “defective” (NOK) based on the thickness uniformity of the zinc layer across the coil width. This application is critical for automotive manufacturing, as inconsistent coating can lead to premature corrosion or mechanical failure in structural components [30,31]. As illustrated in Figure 3, the hardness of zinc coatings varies significantly with composition, ranging from 70 HV for pure zinc to 250 HV for zinc–iron alloys, while the base steel measures approximately 159 HV. This metallurgical variation directly impacts coating quality, as an uneven hardness distribution can lead to defects affecting both corrosion resistance and structural integrity in automotive applications [30].

**Figure 4:**
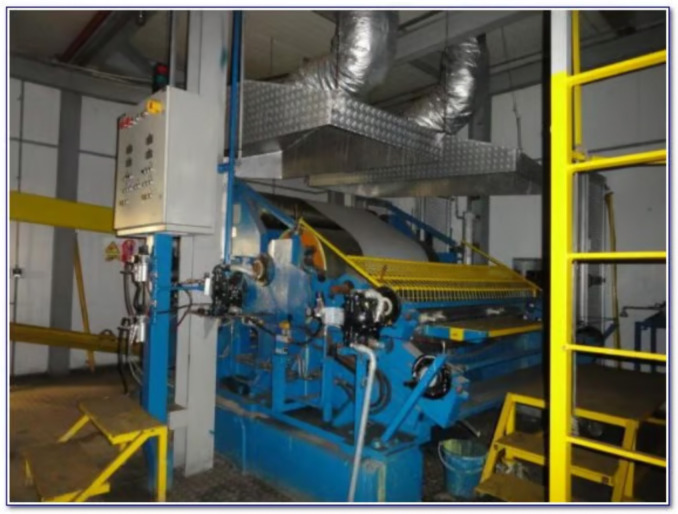
Automated zinc coating thickness inspection system for steel coils.

Current inspection systems employ multiple precision sensors across each coil’s width (Figure 4) and still require human oversight for borderline cases [1]. An automated AI-based solution could significantly improve inspection speed, consistency, and cost-effectiveness. However, the dataset presents two significant challenges: (1) extreme class imbalance, with defective samples constituting less than 3% of all data points, and (2) limited augmentation possibilities, as the spatial patterns of the thickness measurements must be preserved to maintain their diagnostic value. These constraints make this application an ideal test case for comparing DTL and CL approaches under realistic industrial conditions.

**Figure 5:**
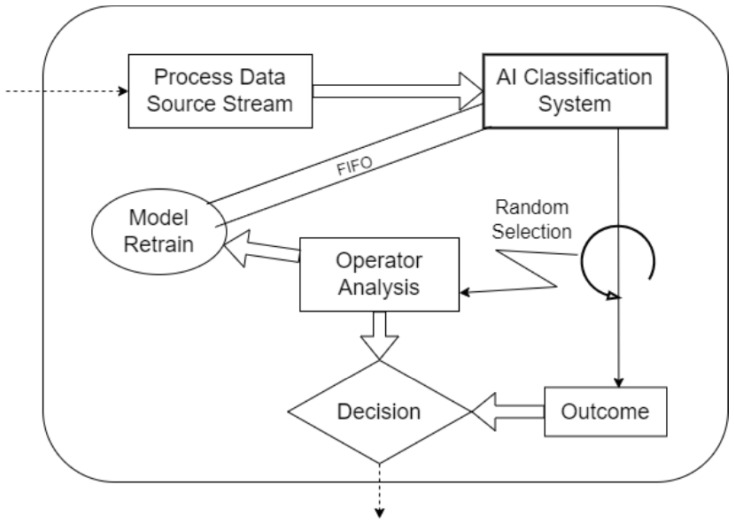
AI classification system flow diagram with human-in-the-loop feedback for continuous learning.

This work directly contrasts DTL and CL under identical conditions on a real industrial dataset. We integrate the full experimental pipeline of a project, including data processing, model training, and evaluation on a hidden gold-standard set, to ensure a fair comparison. Moreover, we introduce an additional perspective by incorporating a human-in-the-loop feedback mechanism in the system design. In practical deployment, the model’s predictions can be monitored and any mistakes like missed defects can be fed back for re-training. We outline how this asynchronous feedback and periodic re-training cycle can sustain model performance over time, which is an additional novelty that strengthens the system’s adaptability beyond the initial static training. The global concept of the configured system is presented in Figure 5, where the system benefits from the asynchronous feedback of the human operator to enrich the classification categories. However, because of the heavily unbalanced categories, the training process includes an embedded factory for data augmentation, looking to keep the training/re-training processes unbiased. The operator’s provided feedback for coils is used to measure the performance of the existing models, while underperformance below a stablished threshold is the signal to request a model re-training, replacing the underperforming models.

This paper presents comprehensive studies comparing DTL and CL in the context of visual quality inspection with extreme class imbalance, proposing a hybrid workflow combining automated learning with expert feedback.

## 2. Literature Review

In recent years, the application of artificial intelligence in industrial quality control has seen significant advancements, particularly in addressing the challenges of imbalanced datasets where defective samples are rare compared to non-defective ones. This section provides a comprehensive review of the current state-of-the-art in Deep Transfer Learning (DTL) and Contrastive Learning (CL) approaches for industrial quality inspection with heavily unbalanced data, especially in scenarios where data augmentation possibilities are limited.

### 2.1. Industrial Quality Control and the Challenge of Data Imbalance

The challenge is further compounded by the limited possibilities for data augmentation in industrial settings. Unlike natural images where rotations, crops, and color adjustments can create realistic variations, industrial defect data often contain spatial patterns and relationships that must be preserved for meaningful analysis [11]. As noted by Semitela et al. (2025) [32], “despite the extensive vision-based deep learning reports for surface defect detection, only a few reports applied this technology to specific industrial products”, indicating a gap in domain-specific applications.

### 2.2. Deep Transfer Learning in Industrial Quality Control

Deep Transfer Learning (DTL) has emerged as a powerful approach to address data scarcity and imbalance in industrial quality control. DTL leverages knowledge from models pre-trained on large datasets and transfers this knowledge to specific industrial tasks with limited labeled data [26,33].

Recent research by Semitela et al. (2025) [32] demonstrated the effectiveness of transfer learning for surface defect detection in heating devices. Their study compared a CNN built and trained from scratch against transfer learning approaches using pre-trained ResNet-50 and Inception V3 architectures. The results revealed that “the pre-trained networks achieved higher accuracies on defect classification compared to the self-built network, with ResNet-50 displaying higher accuracy” [23]. This finding supports the notion that transfer learning can effectively capture subtle defect patterns even with limited training data.

Similarly, Zhao et al. (2025) [34] proposed a cross-machine intelligent fault diagnosis method based on a residual full convolutional neural network (ResFCN) transfer learning model. Their approach effectively exploited “fault-associated general features in the source domain and learned domain-specific patterns that better align with the target domain”. This work is particularly relevant as it addresses the challenge of cross-domain adaptation, where source and target equipment may differ significantly.

The hierarchical nature of deep learning models makes them particularly suitable for transfer learning in industrial applications. As illustrated in [34], “the initial layers of the model extract shallow, general features, while the deeper layers are increasingly focused on learning task-specific features”. This structure allows for effective knowledge transfer across different industrial domains while preserving the ability to adapt to specific defect characteristics.

Several strategies have been developed to enhance DTL performance on imbalanced industrial datasets. One common approach involves careful layer freezing during fine-tuning. Research has shown that freezing a majority of layers, keeping only the last few layers trainable, often yields the best results for smaller models when dealing with imbalanced data [35,36].

Weighted loss functions represent another effective strategy for addressing class imbalance in DTL. By assigning higher weights to minority class samples during training, models can be encouraged to pay more attention to defective samples despite their rarity [37]. This approach has been shown to improve defect recall without significantly compromising precision.

Ensemble methods have also proven effective in industrial quality control with imbalanced data. By combining multiple transfer learning models, each potentially capturing different aspects of defect patterns, overall system robustness can be improved [38]. This approach is particularly valuable in industrial settings where false negatives (missed defects) can have serious consequences.

DTL offers several distinct advantages for industrial quality control applications with imbalanced data. First, it significantly reduces the need for large labeled datasets, which are often impractical to obtain in industrial settings [39]. Second, pre-trained models provide robust feature representations that can capture subtle defect patterns even with limited examples [40]. Third, DTL models typically converge faster during training, reducing computational requirements [41]. However, DTL also faces limitations in industrial applications. Domain shifts between pre-training data (often natural images) and industrial data can reduce transfer effectiveness [42]. Additionally, pre-trained architectures may not be optimized for specific industrial defect types, potentially limiting their ability to capture domain-specific features [43].

In our context of zinc coating inspection, we employ DTL by fine-tuning YOLOv8, a state-of-the-art vision model which has been used in industrial environments for a while [44]. YOLOv8 was originally designed for object detection but can be repurposed for classification with its backbone features [45]. Pre-trained on a massive generic dataset, YOLOv8 provides a strong starting point for our task [45]. We will compare different models of YOLOv8 [46] (Table 1) and explore how many layers to freeze during fine-tuning. Freezing more initial layers preserves more of the general features, which can be beneficial when data are very limited [47]. Indeed, our experiments found that freezing a large portion of the YOLOv8 backbone improved performance for the smaller model variants, which aligns with our intuition for highly imbalanced training data. Overall, DTL provides a simple yet effective way to tackle our problem. By building on pre-trained networks, we aim to achieve high accuracy even with a small number of defect samples.

### 2.3. Contrastive Learning Under Data Imbalance

Contrastive Learning (CL) has emerged as an alternative approach for addressing data scarcity and imbalance in industrial quality control. CL learns an embedding space where similar samples are mapped close together while dissimilar samples are pushed apart, enabling effective feature learning even with limited labeled data [48,49]. Recent research by Schäfer et al. (2024) [50] introduced CLRiuS, a self-supervised Contrastive Learning approach for steel scrap classification that “outperforms existing supervised approaches on the used scrap dataset”. This work is particularly significant as it demonstrates CL’s effectiveness on “intrinsically unordered” industrial images, which differ substantially from the object-centric images typically used in computer vision research.

Similarly, Chen et al. (2024) [51] proposed a self-degraded contrastive domain adaptation (Sd-CDA) framework for industrial fault diagnosis with bi-imbalanced data. Their approach “first pre-trains the feature extractor via imbalance-aware contrastive learning based on a model pruning to learn the feature representation effectively” [51]. This work directly addresses the challenge of imbalanced data in industrial settings, showing that contrastive approaches can be adapted to handle class imbalance. Wu et al. (2024) [52] developed a holistic semi-supervised method for imbalanced fault diagnosis that incorporates an “OOD detection strategy based on contrastive learning”. This approach is particularly valuable for industrial applications where out-of- distribution samples may represent novel defect types or operating conditions not seen during training.

Several specialized techniques have been developed to enhance CL performance on imbalanced industrial datasets. One approach involves careful pair formation strategies that ensure minority class samples are adequately represented in the training process [6,53]. By controlling the distribution of positive and negative pairs, CL models can learn more balanced representations despite underlying data imbalance.

Modified contrastive loss functions have also been proposed to address class imbalance. For example, class-aware contrastive loss assigns different weights to pairs based on their class membership, giving higher importance to minority class samples [6,54,55]. Similarly, supervised contrastive loss incorporates label information to create more informative positive and negative pairs [56].

Multi-reference approaches represent another strategy for handling imbalanced data in CL. Rather than using a single reference image per class, multiple representative examples can be selected through clustering to capture the diversity within each class [57]. This approach is particularly valuable for industrial applications where defect manifestations can vary significantly.

**Figure 6:**
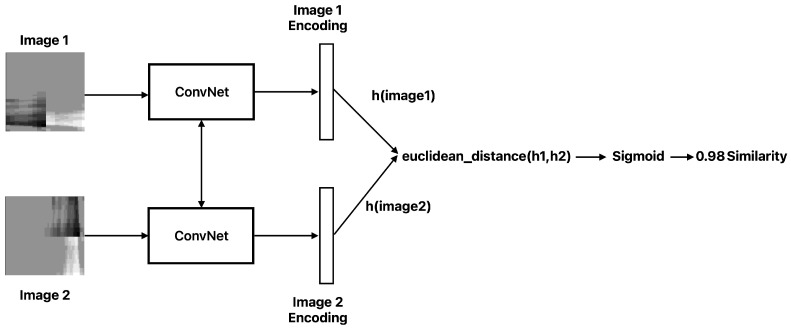
Siamese network architecture for contrastive learning with similarity scoring.

A popular architecture for supervised CL is the Siamese network, which consists of twin subnetworks with shared weights (Figure 6) [18]. Each subnetwork produces an embedding for an input image, and a contrastive loss then brings embeddings of the same class closer while pushing those of different classes apart [15]. Figure 1 illustrates two input images (e.g., one known OK, one test sample) passing through identical CNN encoders, and the distance between their embeddings is computed [58].

Training adjusts the encoder so that the distance is low for image pairs of the same class (OK vs. OK, NOK vs. NOK) and high for mismatched pairs. After training, the model can classify a new image by comparing it to reference exemplar; whichever reference yields the smallest distance indicates the predicted class. This one-shot classification scheme is powerful when we have very few examples of the minority class, since we can make do with even a single prototype image per class [59].

Siamese networks have indeed shown success in industrial scenarios; a study proposed Siamese-RCNet for surface defect detection on textured materials [22]. Their model used a Siamese structure within a Cascade R-CNN detection framework, effectively focusing on subtle anomalies against complex backgrounds [22]. Even with only 20% of the training data labeled, the Siamese-RCNet achieved 96.9% mAP on a surface defect dataset [22], outperforming conventional detectors and greatly reducing the manual annotation required. This demonstrates CL’s ability to extract useful signals from limited data by leveraging unlabeled samples and similarity learning.

CL offers several distinct advantages for industrial quality control with imbalanced data. First, it can effectively learn from unlabeled or partially labeled data, reducing the annotation burden [60]. Second, CL focuses on learning discriminative features through relative comparisons rather than absolute classification, potentially making it more robust to class imbalance [61]. Third, CL can adapt to domain shifts and novel defect types through its focus on feature similarity rather than fixed class boundaries [62,63].

However, CL also faces limitations in industrial applications. The contrastive loss can be challenging to optimize, especially with extremely imbalanced data [64]. Additionally, the performance of CL heavily depends on the quality and diversity of the pairs formed during training, which may be limited in industrial settings with few defect examples [65]. Finally, CL typically requires careful hyperparameter tuning to achieve optimal performance, potentially increasing implementation complexity [66].

### 2.4. Class Imbalance in Quality Inspection

Class imbalance is common in industry since defects are usually rare [67]. In quality inspection, an imbalanced dataset means an algorithm can achieve high overall accuracy by simply always predicting the majority class (always OK), yet completely fail to detect the minority class (the actual defects) [68]. Thus, evaluation metrics beyond accuracy are crucial [3]. We emphasize the use of precision, recall, and F1-score, especially for the minority class, to obtain a realistic assessment of model quality [3]. For instance, a model that catches 90% of defects (high recall) but also falsely flags many good products (low precision) may disrupt production with false alarms. Conversely, a model with high precision but low recall might miss too many defects, defeating the purpose of automation. Our study will report class-wise performance to highlight these trade-offs [29]. Common techniques to handle imbalance include data resampling (oversampling minority or undersampling majority) [6], data augmentation (synthesizing new minority examples) [7], and cost-sensitive learning (using different weights or losses) [6]. In our case, oversampling and augmentation were applied, but within limits. We generated additional NOK training samples through transformations of the few real defect images like slight rotations and noise, but, as noted, data augmentation does not provide enough diversity to meet the learning needs in this task because transformations must be compatible with the mechanical source of defects because of production restrictions. Coating defects have specific patterns that are hard to simulate with generic transforms. Some researchers have used Generative Adversarial Networks (GANs) to create synthetic defect samples and balance the training set [7]. For example, GANs were used to generate unstable (fault) power system states to supplement rare event data [7], and similar approaches have been tested for manufacturing defects. While promising, GAN-generated samples can sometimes be unrealistic or out-of-distribution [7]. In future work, one could explore applying GAN augmentation for coil defects, but discriminators need to implement realistic rules to decide whether specific defects are compatible with production schemas. Currently, this is outside the scope of this paper. Instead, we use DTL and CL to handle imbalance, with DTL providing strong features and CL using pairing and special loss functions.

### 2.5. Comparative Analysis of DTL and CL for Imbalanced Industrial Data

Direct comparisons between DTL and CL approaches for industrial quality control with imbalanced data have been limited in the literature. However, Zhao et al. (2024) [69] provided a comprehensive review comparing transfer learning and self-supervised learning approaches across various domains. Their analysis revealed that while both approaches can effectively address data scarcity, their relative performance depends significantly on the specific application and data characteristics. In terms of accuracy metrics, DTL typically demonstrates superior performance when a suitable pre-trained model is available and the domain shift is not severe [70]. The rich feature representations learned from large datasets often transfer well to industrial defect detection tasks, particularly for visual inspection applications [71]. However, CL may offer advantages in scenarios with extreme data imbalance or when the industrial data differ substantially from available pre-training datasets [72]. By learning directly from the relationships between samples, CL can potentially capture more domain-specific features relevant to the particular industrial application [73]. Computational efficiency also differs between the approaches. DTL often requires less training time as it leverages pre-trained weights, while CL typically involves more complex optimization processes, particularly when forming effective sample pairs [74]. This difference can be significant in industrial deployments where model updates may need to be performed regularly. The suitability of DTL versus CL depends significantly on the specific industrial application and data characteristics. DTL tends to excel in visual inspection tasks where pre-trained models on natural images can provide useful feature extractors [75]. Applications such as surface defect detection, product quality assessment, and component inspection often benefit from transfer learning approaches [32,76,77]. In contrast, CL may be more suitable for industrial applications with unique data characteristics that differ substantially from available pre-training datasets [51,78]. Examples include specialized sensor data, time-series measurements, or highly domain-specific visual patterns that are rarely represented in general image datasets [79]. The degree of data imbalance also influences the choice between DTL and CL. While both approaches can address imbalance, CL’s focus on learning from sample relationships rather than absolute class distributions may provide advantages in cases of extreme imbalance [23,80]. However, DTL with appropriate class weighting and fine-tuning strategies can also perform well in imbalanced scenarios [32,81]. Recent research has explored hybrid approaches that combine elements of both DTL and CL to leverage their complementary strengths. For example, contrastive pre-training followed by transfer learning fine-tuning has shown promise in several domains [82]. This two-stage approach allows models to first learn general feature representations through contrastive learning before adapting to specific classification tasks through supervised fine-tuning. Ensemble methods that combine predictions from both DTL and CL models have also demonstrated improved performance on imbalanced industrial datasets [83]. By integrating the strengths of both approaches, these ensembles can achieve higher accuracy and robustness than either approach alone, particularly for challenging defect types [84,85].

### 2.6. Limited Data Augmentation in Industrial Settings

Data augmentation plays a crucial role in addressing data scarcity and imbalance, but industrial applications face unique challenges in this regard. Unlike natural images where transformations like rotations, flips, and color adjustments can create realistic variations, industrial data often contain spatial patterns and relationships that must be preserved for meaningful analysis [86]. As noted by Semitela et al. (2025) [32], in industrial quality control, “we avoided augmentations that could distort the fundamental spatial pattern, such as large rotations or cut-and-paste, because the coating defect’s signature is subtle”. This constraint significantly limits the range of applicable augmentation techniques, particularly for specialized industrial data. Time-series data from industrial sensors present additional challenges for augmentation. Traditional image-based augmentation techniques are not directly applicable, requiring specialized approaches that preserve temporal dependencies and physical constraints [87]. This limitation is particularly relevant for fault diagnosis applications that rely on sensor measurements rather than visual inspection [88]. Despite these constraints, researchers have developed specialized augmentation techniques for industrial applications. Generative models, including Generative Adversarial Networks (GANs) and Variational Autoencoders (VAEs), have shown promise for creating synthetic industrial data that preserve critical defect characteristics [9,89]. For time-series industrial data, techniques such as window slicing, jittering, and guided warping have been proposed to create meaningful variations while preserving essential signal characteristics [90]. These approaches can be particularly valuable for fault diagnosis applications based on vibration, acoustic, or electrical measurements [91].

The effectiveness of data augmentation varies significantly between DTL and CL approaches in industrial settings. Research has shown that DTL models, particularly those with substantial pre-training, may be less dependent on extensive augmentation as they already possess robust feature representations [92]. However, appropriate augmentation can still improve their performance on minority classes [93].

In contrast, CL approaches often demonstrate greater sensitivity to augmentation strategies, as the formation of effective positive and negative pairs depends heavily on the diversity and quality of available samples [94,95]. As noted by Schäfer et al. (2024) [50], for their contrastive learning approach, “different types of augmentations [were used] to extract the fine-grained structures that are typical for this type of images”, highlighting the importance of domain-appropriate augmentation for CL.

The limited augmentation possibilities in industrial settings may partially explain the performance differences observed between DTL and CL approaches. When augmentation options are constrained by domain-specific requirements, the rich feature representations provided by pre-trained models in DTL can offer advantages over the pair-based learning of CL [96,97].

### 2.7. Human-in-the-Loop (HITL) in Quality Inspection

Given that data imbalance in production is not static—new defect types may appear over time or more samples can be collected—human-in-the-loop (HITL) strategies have gained attention for maintaining model performance [98,99]. In industrial settings, a common practice is to deploy an initial model and then continuously update it with feedback from human inspectors or operators [100]. For example, an automated inspection system might flag uncertain or novel defect cases for manual review, and those reviewed cases (with confirmed labels) are fed back into the training pipeline during periodic re-training. This adaptive loop can gradually improve the model’s defect recognition capability and address drift in the data distribution. Recent work in imbalanced learning supports the efficacy of targeted data augmentation via active feedback. Liu et al. (2023) [81] introduced a Transfer Learning Classifier (TLC) with an active sampling module that queries additional samples of under-represented classes to improve balance [81,101]. Such active learning approaches, where the model intelligently selects new data points for human labeling, have been shown to significantly boost minority class accuracy in imbalanced image classification [102,103]. In the context of defect inspection, a “balanced active learning” strategy might involve preferentially sampling images from the production line that the model is least confident about (often potential defects) and having an expert label them [102]. This ensures that with each re-training cycle, the data skew is slightly less extreme and the model is exposed to a broader variety of defects. Furthermore, HITL can help catch edge cases that the initial training data did not include—for instance, a new type of surface flaw that triggers a false negative [104]. By incorporating these cases through adaptive re-training, the system remains robust. Some advanced frameworks even include online learning components that update the model on-the-fly using confirmed defect instances, though care must be taken to avoid catastrophic forgetting of older classes. Overall, human-in-the-loop and adaptive learning paradigms are increasingly recognized as practical necessities for sustaining high model performance in quality control systems. They complement the algorithmic solutions by addressing the data side of the imbalance problem: over time, the model’s knowledge of the minority class is improved not just by synthetic tweaks, but by real new examples curated with expert oversight.

### 2.8. Comparative Studies of Deep Transfer Learning Versus Contrastive Learning

Although both Deep Transfer Learning (DTL) and Contrastive Learning (CL) have demonstrated strong performance in isolation, only a few works have directly pitted them against one another under the same conditions. In the medical imaging domain, Zhao et al. (2024) [69] carried out a rigorous comparison using two popular CNN backbones—Xception and ResNet—trained under transfer learning and supervised self-supervised (contrastive) regimes, and then fine-tuned on four small datasets: two color collections (Kvasirv2 endoscopy and EyePacs fundus images) and two grayscale sets (BusI histopathology and Chest CT scans). They reported that transfer learning achieved higher accuracy on color tasks (96.4% on Kvasirv2, 92.1% on EyePacs), whereas contrastive SSL outperformed on grayscale (97.2% on Chest CT, 90.0% on BusI). Importantly, they showed that a double fine-tuning protocol—first on a related source, then on the target—further mitigates domain shift without extra pre-training data [69,105]. In small-component assembly inspection, Shiwen Zhao et al. (2024) [77] proposed a two-stage pipeline combining supervised contrastive pre-training with a Siamese network on unlabeled assembly images, followed by transfer learning fine-tuning on a limited labeled set of correctly versus mis-assembled parts. Their Advanced Engineering Informatics case study on flex-head ratchet wrench components demonstrated that this hybrid CL to TL approach outperformed a pure transfer baseline by over 5 percentage points in defect classification accuracy, while requiring up to 40% fewer labeled examples. In the context of metal surface defect detection, Mahe Zabin et al. (2023) [106] introduced a self-supervised contrastive framework that leverages large unlabeled steel images to learn robust embeddings, then fine-tunes them on the NEU-CLS dataset.

Despite using a lightweight encoder, their model achieved 97.78% classification accuracy—surpassing several transfer learning baselines—and did so with fewer trainable parameters. This study underscores CL’s ability to extract discriminative features from unlabelled data in highly unbalanced defect scenarios. Shifting to power systems, Jinman Zhao et al. (2024) [7] proposed a **Contrastive-Active Transfer Learning** method for real-time transient stability assessment in electricity grids. Their approach uses an offline contrastive pre-training phase to enhance representation under severe class imbalance (stable vs. unstable events), then an active online transfer step to adapt to new operating conditions. This two-stage scheme improved unstable-event detection accuracy by approximately 12% over standard TL, while cutting required update samples by half. Finally, at the survey level, Zehui Zhao et al. (2024) [69] performed a broad review across multiple domains—medical imaging, industrial vision, and beyond—summarizing definitions, applications, strengths, and limitations of both transfer and self-supervised pre-training. They concluded that transfer learning generally excels when moderate labeled data exist, whereas self-supervised methods outperform under extreme label scarcity. Moreover, they advocate double fine-tuning and hybrid CL to TL strategies as best practices to bridge performance gaps and manage domain mismatch.

### 2.9. Research Gaps and Future Direction

Despite significant advances in both DTL and CL for industrial quality control, several important research gaps remain. First, direct comparative studies between these approaches under identical conditions with industrial data are scarce, making it difficult to draw definitive conclusions about their relative merits [69,77,107]. Most studies focus on either DTL or CL in isolation, with different datasets and evaluation metrics. Second, the impact of extreme class imbalance, particularly with ratios exceeding 1:100 between normal and defective samples, has not been thoroughly investigated [108]. Such extreme imbalance is common in high-quality manufacturing environments but presents significant challenges for model training and evaluation. Third, standardized benchmarks for industrial quality control with imbalanced data are lacking, hindering direct comparisons between different approaches [109]. Unlike fields such as natural image classification or object detection, industrial quality control lacks widely accepted benchmark datasets that reflect real-world challenges. Fourth, domain-specific challenges in industrial quality control, such as the limited augmentation possibilities discussed earlier, are often not adequately addressed in the general machine learning literature [110]. These constraints can significantly impact the applicability of theoretical advances to practical industrial deployments. Several promising research directions are emerging to address these limitations. Human-in-the-loop approaches that combine automated inspection with expert feedback show potential for continuously improving model performance with minimal additional labeling [111]. This approach is particularly valuable for industrial settings where domain experts are available but their time for annotation is limited. Continual learning frameworks that allow models to adapt to evolving industrial environments without catastrophic forgetting represent another important direction [112,113]. As manufacturing processes and defect patterns change over time, models must be able to incorporate new knowledge while retaining previously learned patterns. Multi-modal approaches that combine different data sources, such as visual inspection, sensor measurements, and process parameters, offer opportunities for more robust defect detection [114,115]. By integrating complementary information, these approaches can potentially overcome the limitations of single-modal methods, particularly for complex industrial systems. Explainable AI for industrial quality inspection is gaining importance as regulatory requirements and safety considerations demand transparent decision-making [116]. Methods that not only detect defects but also provide interpretable explanations for their decisions can increase trust and adoption in critical industrial applications.

### 2.10. Novelty and Contributions of the Current Study

In response to the key research gaps identified in the literature, this study presents several novel and impactful contributions to the field of industrial quality control using machine learning. The most prominent of these is the first comprehensive, head-to-head comparison between Deep Transfer Learning (DTL) and Contrastive Learning (CL) in the context of industrial quality inspection with extreme class imbalance. While earlier research has explored these techniques in isolation, this study uniquely evaluates both approaches under identical conditions using real-world data from galvanized steel coil inspections. This fills a critical void in the comparative literature and offers actionable insights for practitioners working with similar industrial datasets.

Another significant contribution lies in the evaluation of model performance under realistic industrial constraints, particularly where data augmentation is limited. Due to the necessity of preserving spatial patterns in zinc coating measurements, typical augmentation strategies cannot be applied. This constraint, although common in real-world manufacturing, is rarely considered in academic studies. By incorporating this challenge, this study enhances the relevance and applicability of its findings to industrial settings.

The research also proposes a novel human-in-the-loop feedback mechanism designed to maintain model performance over time. Through asynchronous feedback and periodic re-training cycles, the system adapts to evolving production environments. This represents a meaningful advancement in the development of adaptive learning systems, addressing the dynamic nature of real-world manufacturing conditions where data distributions may shift over time.

Methodologically, the study introduces a rigorous cross-validation strategy tailored for extremely imbalanced industrial datasets. By combining this approach with evaluations on a balanced, gold-standard test set, it ensures a fair and robust assessment of model effectiveness. This contribution establishes a replicable framework for evaluating machine learning models in similarly challenging industrial scenarios.

Lastly, the study provides practical implementation guidance, bridging the gap between academic research and industrial deployment. It includes recommendations for computational efficiency, system integration, and deployment strategies tailored to manufacturing environments. These insights empower engineers and quality managers to make informed decisions when applying DTL or CL methods in their production pipelines.

Overall, by tackling both theoretical and practical aspects of learning under extreme imbalance in industrial contexts, this study not only deepens academic understanding of DTL and CL techniques but also offers valuable tools and frameworks for real-world implementation in quality control systems.

## 3. Methodology

### 3.1. Case Study: Galvanized Steel Coil Dataset

Our case study centers on galvanized steel coils used in the automotive industry. Each coil is coated with zinc to prevent corrosion, and quality is determined by the uniformity of the zinc coating thickness. Currently, the plant uses an array of X-ray sensors across the width of the steel strip to measure coating thickness at multiple points.

Some preprocessing activities are required here, because tensor processing frameworks such as Tensorflow or Pytorch require embeddings of length variable samples to fit in the tensor size [117]. In many industrial applications, product length is variable, such as coils, bars, beams, etc. However, in our application, since decisions are made at the item level, we decided to create a fixed number of chunks per item, each with potentially different length when compared through items. Inside each chunk, interesting variables are averaged to represent condensed behavior, while standard deviation accounts for inner variability. In our application, coil length was divided in 264 chunks according the product’s internal characteristics.

**Figure 7:**
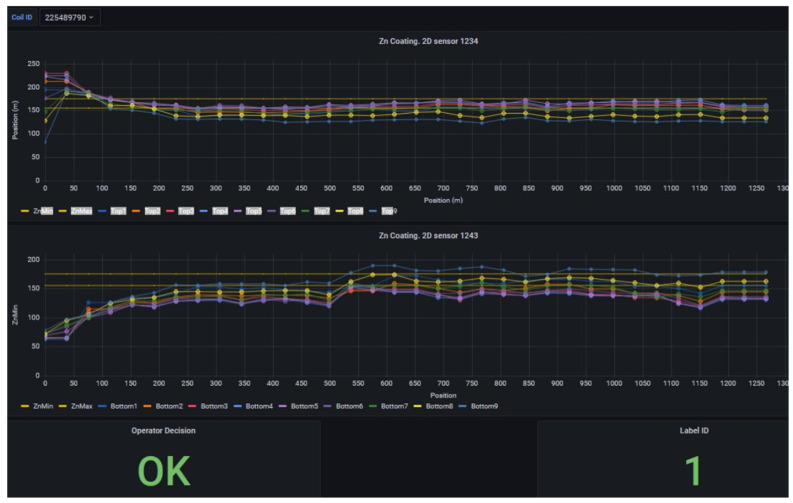
Zinc coating thickness measurement matrix with quality labeling (OK/NOK).

In our dataset, each coil’s coating profile is represented as a matrix of 264 × 18 measurements. Here, 264 corresponds, as already said above, to sections along the length of the coil, and 18 corresponds to 9 sensors on the top side and 9 on the bottom side of the strip, responsible to measure coating thickness. This matrix can be shown as a 264 by 18 pixel image, where intensity reflects thickness, similar to a heatmap of zinc coating. As can be seen in Figure 7, each matrix (image) is labeled as OK if the coil met quality specs or NOK if it was unacceptable due to thin spots or other coating defects. An entire coil receives an NOK label if a relevant section falls below the threshold; in practice, this classification could be made per section as well, but here we treat each sensor matrix as an instance labeled OK/NOK.

To address potential data shifts inherent in industrial settings, our approach incorporates strategies that align with domain adaptation principles. Specifically, we utilize Deep Transfer Learning (DTL) with a pre-trained model (YOLOv8), which is fine-tuned on our dataset of galvanized steel coils. This fine-tuning process adapts the model from a general domain, such as ImageNet, to our specific target domain, effectively bridging the gap between different data distributions. Additionally, our system includes a human-in-the-loop feedback mechanism where operators provide feedback on model classifications. This feedback is used to assess model performance and trigger re-training when necessary, ensuring that the models adapt to any shifts in the data distribution over time. These methods, while not explicitly labeled as domain adaptation, inherently address the challenges of varying data distributions in real-world industrial environments.

It is important to highlight that the decision process is not as simple as matching the thresholds or not; it requires considering where the failure happens, how big it is, which customer tolerance is enforced, and market conditions. Therefore, decision criteria are not easy to represent via a mathematical expression, which makes the system suitable for an integrated case-based learning process.

The dataset contains 4542 such images for training/validation, collected from production process over time and labeled by operators. As expected, it is highly imbalanced, as only a few percent of these are NOK. To evaluate generalization, we set aside a gold standard test set of 60 images including 30 OK and 30 NOK. These were selected from a later time period, representing unknown data not seen during model development. The fifty–fifty balance in the gold set ensures that evaluation clearly reflects defect detection capability, and the balanced test F1-score is effectively the harmonic mean of recall and precision on defects. The models were not tuned on this gold set and it was strictly used for final performance reporting.

**Figure 8:**
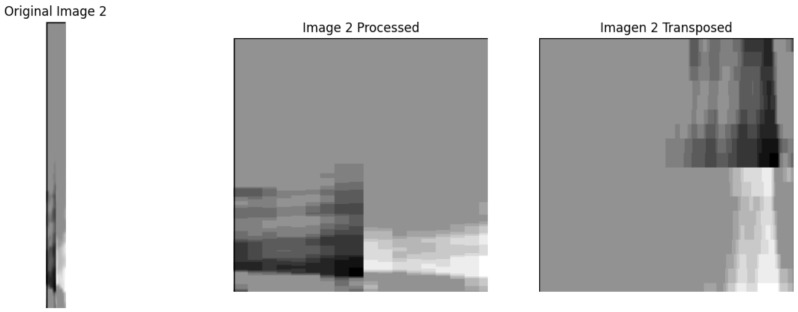
Visualization of preprocessing steps transforming matrix data (264 × 18) into square images (264 × 264 pixels) for YOLOv8 classification.

Before feeding the data to models, we performed some data augmentation and transformations. The process of transforming the original sensor matrices into square images suitable for YOLOv8 classification is explicitly illustrated in Figure 8.

Each 264 × 18 matrix was normalized considering the global limits from the population, and converted to a grayscale image. We also experimented with resizing or reshaping the matrices into larger square images. In one experiment, we transformed each matrix into a square image by interpolation and tiling to preserve spatial relationships in a more isotropic format. However, we used the original matrix shape for our main results. Data augmentation was mainly applied to the training set, focusing on the minority class (NOK) to increase its presence. Augmentations included small rotations, since the orientation of the coil image is fixed and rotation simulates slight sensor calibration differences, as well as flips and Gaussian noise injection. We avoided augmentations that could distort the fundamental spatial pattern, such as large rotations or cut-and-paste, because the coating defect’s signature is subtle. Despite augmenting NOK images by several folds, the dataset remained imbalanced. We did not undersample OKs because that would throw away valuable data. Instead, we relied on robust modeling techniques such as DTL and CL, along with appropriate loss functions, to handle the learning process.

For model evaluation, we adopted a cross-validation strategy in addition to the gold-standard test. The 4542 images were split into training and validation folds using stratified 5-fold cross-validation, ensuring the tiny NOK ratio was preserved in each fold. Each model was trained and validated on 5 folds, and this entire 5-fold training was repeated 10 times with different random fold splits to ensure stability. This procedure, consisting of 5-by-10 runs, provided a distribution of performance metrics, from which we selected the model configuration with the highest average validation F1-score. This rigorous approach mitigates any variance due to training data ordering or initialization, which is important given the potential volatility when minority samples are so few. Once the best configurations for DTL and CL were identified via cross-validation, we re-trained each on the full 4542-sample set (excluding the gold 60) and then evaluated them on the 60-image gold set once. The gold set performance is reported as the definitive result, simulating how the model would perform on truly unseen production data.

**Figure 9:**
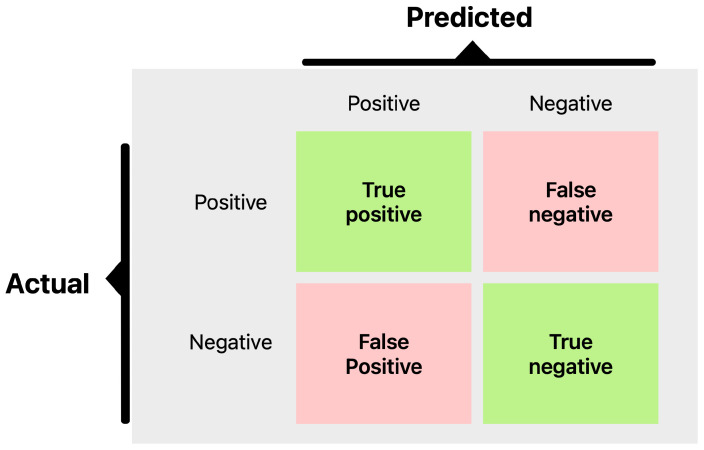
Confusion matrix for zinc coating quality classification (OK/NOK).

The metrics we focus on are per-class precision, recall, F1-score, overall accuracy, and ROC-AUC. Precision for the NOK class tells us what fraction of predicted defects were actual defects; low precision indicates many false alarms. Recall for NOK shows what fraction of true defects were detected; low recall means many were missed. F1-score combines precision and recall into a single value for defects. We also track the F1-score for the OK class and the macro-F1. ROC-AUC provides a threshold-independent measure of class separability. However, in cases of extreme imbalance, AUC can be overly optimistic because it gives significant weight to true negatives, which are abundant in the OK class. Therefore, we place more emphasis on the F1-score and the confusion matrix (Figure 9. During training, we monitored validation F1, both macro and specifically for NOK, and applied early stopping when necessary to prevent overfitting.

### 3.2. Deep Transfer Learning Model (DTL)

For deep transfer learning, we chose the YOLOv8 family of models. YOLOv8, introduced in 2023, is the latest in the YOLO (You Only Look Once) series known for fast and accurate object detection. We selected YOLOv8 variants due to their proven efficiency and robust performance in industrial defect detection scenarios. YOLOv8 combines convolutional neural networks (CNNs) with spatial attention mechanisms, which are highly effective at extracting features from images, such as edges, textures, and shapes. Spatial attention enhances the model’s focus on the most relevant image parts for detection tasks, significantly improving accuracy and robustness. Furthermore, YOLOv8 models, pre-trained on the comprehensive ImageNet dataset, offer variants ranging from small (nano) to extra-large, differing in parameters and computational complexity. Smaller models like YOLOv8s generally offer a better trade-off between model complexity and generalization capability, particularly when dataset size and computational resources are limited. Therefore, the choice of YOLOv8s was validated through extensive cross-validation tests, which demonstrated optimal results in terms of accuracy, precision, recall, and F1-score by carefully balancing the number of frozen layers. Freezing the majority of initial layers effectively retains pre-trained generic features and prevents overfitting, thereby leveraging robust learned representations while enabling task-specific fine-tuning through the last layers. Although our task is classification (OK vs. NOK), we can exploit YOLOv8’s powerful backbone, which was pre-trained on a large-object detection dataset. The intuition is that YOLOv8’s backbone has learned rich features useful for identifying textures and anomalies, which can be repurposed. We utilized the YOLOv8 classification mode, which essentially removes the detection head and uses the final global pooling layer for class prediction.

**Figure 10:**
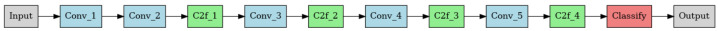
YOLOv8 architecture for classification task with scalable variants.

In Table 1, the comparison has been detailed for five pre-trained YOLOv8 variants: n, s, m, l, and x (nano, small, medium, large, and extra-large). These differ in network depth and width, and correspondingly in the number of parameters (from YOLOv8n with almost 3 million params to YOLOv8x with almost 68 million). Figure 10 shows the YOLOv8 architecture highlighting the different structural blocks utilized during fine-tuning for this specific classification task. Smaller models might generalize better (less overfit risk), but larger models might capture more nuanced patterns if they do not overfit [24]. We therefore treated the model size as a hyperparameter to select via cross-validation.

**Figure 11:**
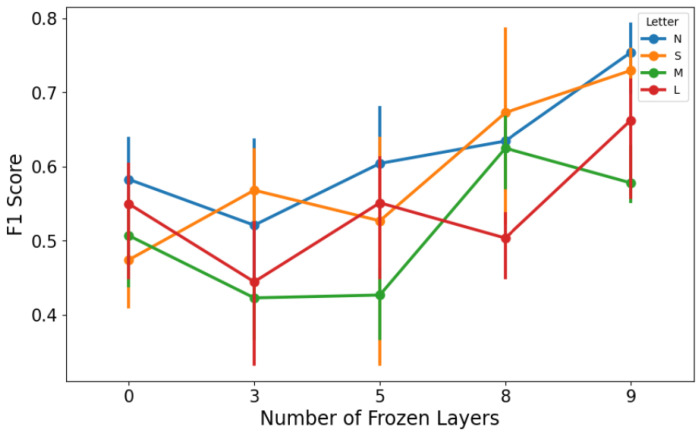
Impact of different numbers of frozen layers on YOLOv8 model performance, highlighting optimal fine-tuning configuration at seven frozen layers.

Another important hyperparameter in transfer learning is the number of layers to freeze during fine-tuning. Freezing means keeping the pre-trained weights fixed (especially in early layers) so that only some top layers and the classification head are re-trained on new data [118]. Freezing can prevent catastrophic forgetting of generic features and reduce overfitting when new data are few. However, freezing too much might limit the model’s ability to adapt to new task-specific features [119]. YOLOv8’s architecture can be viewed as 10 major layers, with each grouping some convolutional blocks. We tried freezing none for full fine-tuning, freezing half, and freezing most layers to see what yields the best validation performance. We found that freezing a majority of layers, keeping only the last 2–3 layers trainable, gave the best results for the smaller YOLOv8n and YOLOv8s models. The impact of freezing different layers during fine-tuning on model performance is explicitly shown in Figure 11, illustrating how model generalization is enhanced by leveraging pre-trained features. This matches observations that transferring to a task with very limited data often benefits from more freezing, thus relying on the robustness of learned features. For the larger models, we could fine-tune more layers without overfitting, but they did not necessarily outperform the smaller ones on F1. Ultimately, the best DTL model was YOLOv8s with 7 of 10 layers frozen, where only the last 3 layers and the output layer were fine-tuned. This model had a good balance of bias and variance for our data.

Training of the DTL model was carried out using a standard cross-entropy loss on the OK/NOK predictions. We used an Adam optimizer [120] with a learning rate in the range 10^−3^ to 10^−4^ (tuned per model size). Training lasted at most 50 epochs per fold, with early stopping if validation loss did not improve for 5 epochs. Due to the class imbalance, we also tried weighting the loss for the NOK class higher to force the model to care about NOK. A weight of 5:1 (NOK:OK) was applied based on the inverse class frequency. This helped improve defect recall slightly in the baseline CNN. For YOLOv8, it also helped to a degree, though the pre-trained features already gave it a strong starting point to recognize NOK. We also monitored the training to ensure the model was not simply predicting all OK, as the weighted loss and our evaluation strategy, which rewards detecting NOK, guard against that.

**Figure 12:**
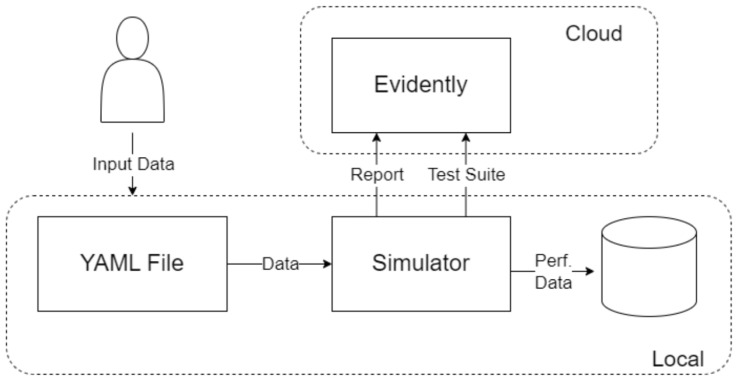
AI model monitoring and performance validation pipeline.

One more technique that was applied was ensemble averaging. After cross-validation, we had several YOLOv8 models from different random initializations or folds that performed well. We ensembled the top 3 by averaging their prediction probabilities for the test set. Ensembling often improves stability and performance, especially to smooth out any quirks one model may have. In our case, the ensemble of three YOLOv8s models yielded a slight boost in gold-set F1 (about +1%). For simplicity, though, we will mainly report the single best model’s performance, noting that ensemble could be used in deployment for extra safety. The complete architecture of the implemented Deep Transfer Learning system, including data preprocessing and training stages, is summarized in Figure 12.

### 3.3. Contrastive Siamese Model (CL)

For the Contrastive Learning approach, we built a Siamese network tailored to binary classification (OK vs. NOK). The architecture consists of two identical CNN branches that merge at the end. In our implementation, Contrastive Learning is not used as a separate feature representation stage followed by classifier fine-tuning. Instead, the Siamese network directly integrates feature learning and classification by producing similarity scores that are used for decision-making. This approach leverages the strengths of CL in learning robust feature representations while ensuring efficiency in handling limited labeled data, a common challenge in industrial settings.

We based the CNN encoder on a smaller convolutional network with 4 convolutional layers followed by a couple of dense layers, which was sufficient to encode the 264 × 18 images. This was designed somewhat empirically, starting with a simple LeNet style network and increasing the depth until performance plateaued. Notably, our Siamese encoder ended up with 10 convolutional layers as the best configuration from simulation studies, indicating a fairly deep network was needed to capture the necessary features. This matched the finding for the baseline CNN that 10 convolutional layers yielded the highest F1.

The selection of these encoder architectures was based on their proven performance in the Deep Learning phase of our research, where they demonstrated superior classification and feature extraction capabilities for our steel coil dataset. Specifically, we prioritized CNN architectures that had already shown effectiveness in direct classification tasks, ensuring consistency and efficiency across the project. This choice allowed us to leverage established models rather than starting from scratch, avoiding redundant testing of less effective architectures.

During training, the Siamese network was fed pairs of images. We formed pairs in two categories. Pairs of OK and OK or NOK and NOK were labeled as match, meaning they belonged to the same class, while OK and NOK pairs were labeled as non-match, indicating different classes. We assigned the label 0 to matching pairs and 1 to non-matching pairs, though this convention could be reversed depending on the implementation. The network outputs a similarity score between 0 and 1. For training, we used a contrastive loss function, which was essentially a binary cross-entropy applied to the output, with the target being 0 for same-class pairs and 1 for different-class pairs. We also experimented with a margin-based contrastive loss, similar to classic Siamese training, where the network tried to enforce a distance smaller than a set margin for same-class pairs and larger than the margin for different-class pairs. While our approach primarily utilizes a binary cross-entropy loss applied to similarity scores for its simplicity and effectiveness, we acknowledge the existence of advanced contrastive loss functions specifically designed for imbalanced datasets, such as the Asymmetric Contrastive Loss (ACL) of Vito and Stefanus from 2022 [6]. ACL modifies the standard contrastive loss to assign higher weights to minority class pairs, which could be particularly beneficial in our scenario where defective (‘NOK’) samples are significantly fewer than acceptable (‘OK’) samples. However, in our preliminary experiments, we found that the simpler binary cross-entropy loss performed comparably to more complex losses, including margin-based contrastive loss, while offering easier interpretation and implementation. This allowed us to focus on other critical aspects of our model, such as the multi-reference design, which proved essential for handling variability in defect manifestations. The results were comparable, so we report the simpler cross-entropy formulation for clarity.

A challenge in using Siamese networks on an imbalanced dataset is generating a meaningful training pair distribution. Since there are far more possible OK-OK pairs than NOK-NOK pairs, we addressed this through careful pair sampling. In each training epoch, we randomly sampled an equal number of same-class pairs from both OK and NOK images. To achieve balance, if we define N_pos as the number of NOK images, we generated approximately N_pos squared pairs of NOK-NOK by pairing each NOK image with others, allowing repetitions as needed. This ensured parity with the OK-OK pairs, where OK images were far more abundant but we randomly selected a subset for pairing. For OK-NOK pairs, we ensured each NOK image was paired with many different OK images to maximize the utilization of all NOK samples. The resulting training set of pairs was much larger than the original image count and roughly balanced between match and non-match pairs. This approach guaranteed the Siamese model encountered as many NOK-involving pairs as OK pairs, effectively mitigating bias. The pair generator created all unique combinations each epoch, incorporating some randomness to ensure the model consistently encountered diverse comparisons throughout training.

After training the Siamese network on pair similarity, we use it for classification in the following way. For a given test image, we compare it against two fixed reference images—one representing OK and one representing NOK—through the two branches of the network. This process produces two similarity scores. We then classify the test image based on which reference image it shows higher similarity to, meaning whichever class reference it is closer to in the embedding space. Initially, we selected one representative OK image and one representative NOK image as references. These reference images could be determined by finding the centroid images of each class cluster from the training samples. In practice, we applied K-means clustering to each class and used the cluster centroids as reference images to capture some natural variability within each class. The approach can be extended to use multiple reference images per class. One implementation would involve running multiple Siamese networks in parallel, with each network comparing the test image to a different prototype. In our specific implementation, we achieved a similar effect by averaging the similarity scores between the test image and several reference NOK images as well as several reference OK images. These multiple references were selected through clustering. This multi-reference approach significantly improved classification robustness since defects can appear in various forms, and no single NOK reference image would be similar to all possible defect variations.

A natural question arises regarding why we did not simply use a standard classifier such as a small CNN for direct OK/NOK classification. For comparison, we did implement a baseline CNN classifier without contrastive loss. While this baseline achieved good validation accuracy, it struggled with either missing many NOK cases or generating excessive false alarms depending on the threshold setting. In contrast, the contrastive Siamese approach was specifically designed to distinguish classes through similarity metrics, with its architecture inherently better suited to handle class imbalance.

**Figure 13:**
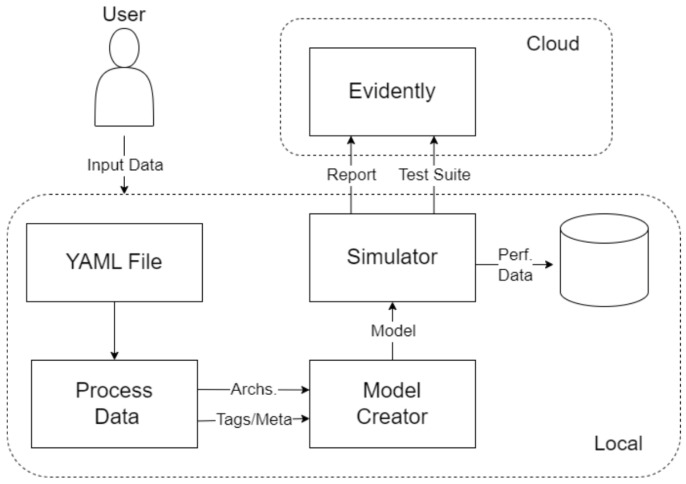
End-to-end contrastive learning system for zinc coating quality inspection.

In practice, our Siamese network demonstrated stable training and achieved near-perfect separation of pair types during training. However, its absolute performance on validation and test sets ultimately fell short of the transfer learning model’s results, as we will discuss later. This performance gap likely stems from several factors. The Siamese network, trained from scratch without external data, had to learn all relevant features solely from our limited training images. While pair generation creates more training combinations, it does nor actually produce new features—it simply highlights differences between existing ones. By comparison, a pre-trained network begins with a rich foundation of learned features like edge detection and gradient recognition, which may prove essential for identifying subtle coating inconsistencies that our limited dataset could not fully capture. Figure 13 illustrates the complete Contrastive Learning framework and its workflow, detailing pair formation, embedding extraction, and similarity evaluation.

### 3.4. Implementation and Tools

**Figure 14:**
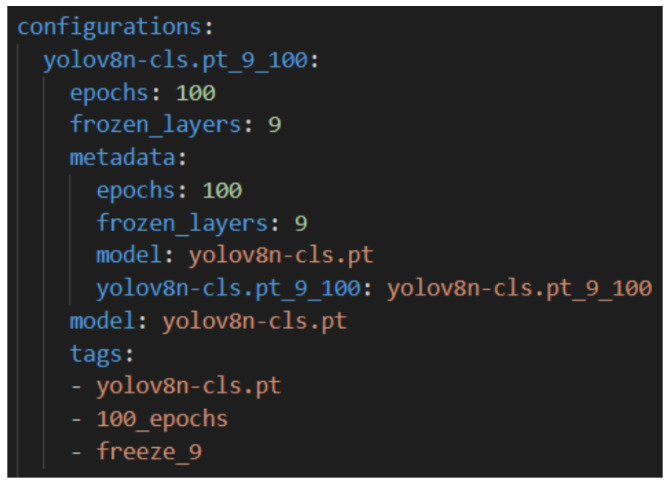
Example YAML configuration file used to define the YOLOv8 training setup.

We implemented both approaches in Python using PyTorch as our deep learning framework. For the YOLOv8 models, we leveraged the Ultralytics framework, which provides PyTorch-compatible weights pre-trained on ImageNet for all YOLOv8 variants from the smallest (n) to the largest (x). An example YAML configuration file used to systematically define YOLOv8 training setups is illustrated in Figure 14.

**Figure 15:**
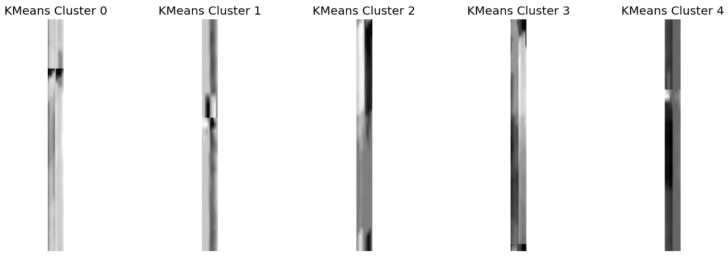
Representative images selected by KMeans clustering for CL reference points.

The Siamese network architecture was custom-built directly in PyTorch. Representative images selected by KMeans clustering for the OK class are clearly shown in Figure 15, illustrating examples used as references in CL.

**Figure 16:**
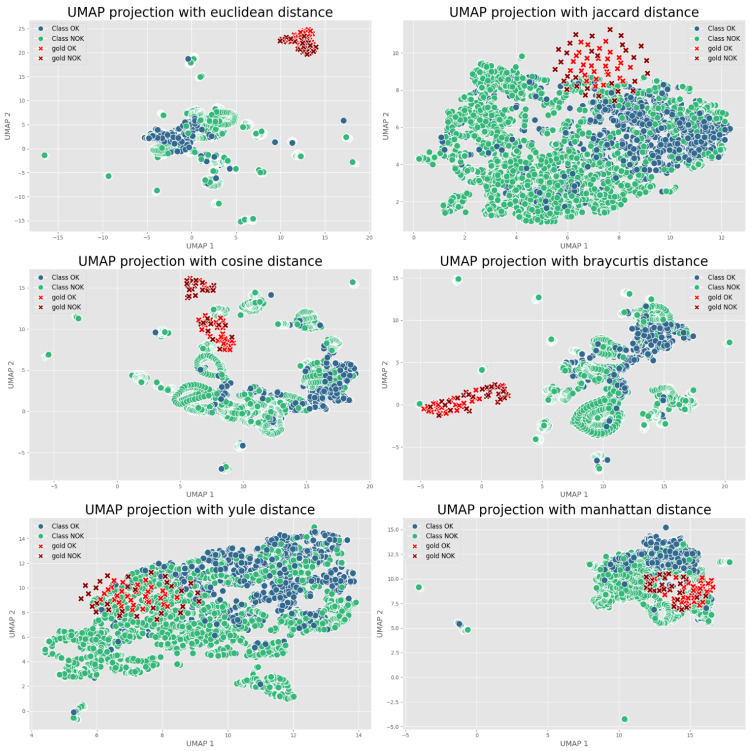
Comparison of clustering results using various distance metrics to select optimal references for CL.

All training occurred on a workstation equipped with an NVIDIA RTX 3080 GPU. Training times varied significantly between approaches. The YOLOv8 models completed training quickly, with smaller variants finishing in just 2–3 min per cross-validation fold and even the largest YOLOv8x model completing within 10 min. This efficiency stemmed from the small input image size and our implementation of early stopping. In contrast, the Siamese network required approximately 15 min per fold due to its pair-based training approach—while architecturally simpler than YOLO, processing image pairs effectively doubled the computational load. We generated an extensive set of training pairs to ensure robust learning. Figure 16 visually compares different clustering distance metrics used for selecting optimal reference images in Contrastive Learning.

For performance monitoring and analysis, we integrated the Evidently AI platform throughout our experiments. This allowed us to systematically track model metrics across all cross-validation folds and compare prediction distributions between training data and our gold standard test set, helping identify any potential performance drift or data distribution shifts. To maintain experimental rigor, we applied identical cross-validation splits to both methodologies. We also kept all preprocessing and augmentation steps consistent across approaches, with the sole exceptions being the fundamental architectural differences and their respective loss functions. This careful control of variables enabled us to isolate and properly evaluate the comparative impact of Deep Transfer Learning versus Contrastive Learning for our specific application.

## 4. Experimental Setup and Integration of Provided System

This section explains how the experiments were carried out to carefully compare Deep Transfer Learning with different YOLOv8 versions and Contrastive Learning using Siamese networks. The process followed the CRISP-DM method, which is commonly used in data mining projects. We will now go through the steps for preparing the data, training the models, tuning their settings, and checking how well they performed. Finally, we introduce the idea of adding human feedback to make the system more practical for real-world use.

### 4.1. Data Preparation

The data came from sensors that measured zinc coating thickness on steel coils. These readings were transformed into structured, image-like matrices with a size of 264 × 18. At first, we tested two different preprocessing setups, each using different scaling methods and sensor channels. Since both gave similar results during validation, we chose one consistent setup for all experiments. The dataset was split into five parts for cross-validation. We also prepared a separate gold-standard test set with 60 images. This set stayed untouched until the final evaluation, making sure the results truly reflect how the system would perform in real-world conditions.

### 4.2. Model Training and Simulation Platform

To properly test how both Deep Transfer Learning and Contrastive Learning perform, we set up a well-organized experimentation platform. It was built around customizable YAML files, which made it easy to run automated tests across a wide range of model settings and hyperparameters. This setup also included full logging, so we could track all the results clearly and consistently.

### 4.3. Deep Transfer Learning

**Figure 17:**
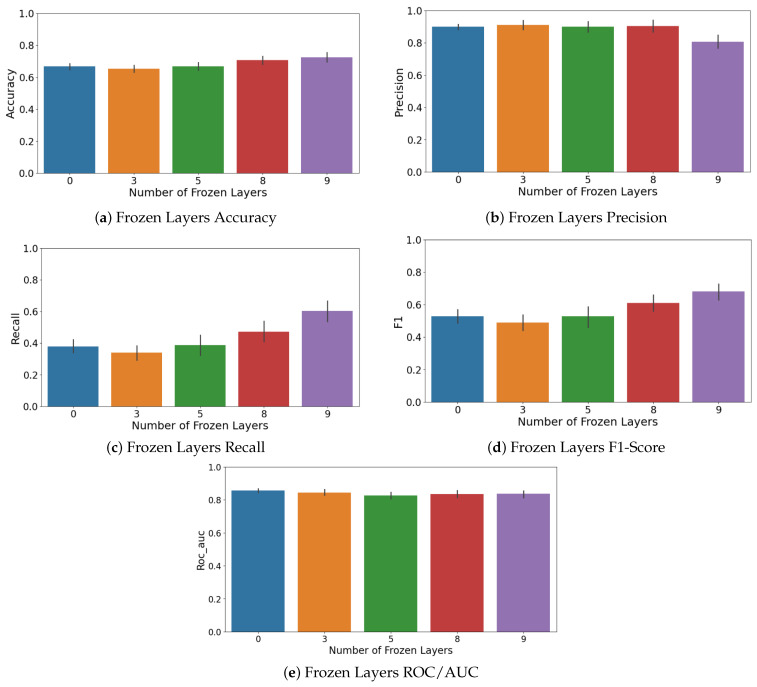
Pre-trained YOLO metrics comparison with frozen layers.

For the Deep Transfer Learning experiments, as already said, we used several versions of the YOLOv8 model released by Ultralytics. To clearly isolate and quantify the benefits of pre-trained weights, we conducted a comparative analysis assessing different configurations of the YOLOv8 model by varying the number of frozen layers during fine-tuning. This comparison evaluated models with 0, 3, 5, 8, and 9 layers frozen. Results indicated that freezing more layers (retaining pre-trained weights) significantly improved key metrics such as accuracy and recall (see Figure 17a–d). Specifically, accuracy improved as more layers were frozen, peaking when nine layers were frozen, suggesting that preserving general pre-trained features enhances stability and classification consistency. Although precision peaked at fewer frozen layers (three layers), the overall optimal performance (F1-score) was achieved with nine frozen layers due to substantial recall improvements, clearly demonstrating the advantage offered by pre-trained knowledge embedded within YOLOv8 models.

These included the n, s, m, l, and x variants, each one growing in size and complexity from about 3 million parameters up to around 68 million. One of the most important things we looked at was how many of the model’s layers should be kept fixed during fine-tuning. This step, known as freezing, helps avoid overfitting, especially when working with smaller datasets. For every version of YOLO, we tested freezing between zero and nine layers, and ran these setups across different cross-validation splits. In total, this gave us 50 unique model combinations. To judge which model performed best, we focused on the F1-score for the less common class in our data, the defective cases. Out of all the models, the YOLOv8s version with seven frozen layers gave us the best and most consistent results. This setup struck a strong balance between keeping the benefits of pre-training and allowing the model to adapt to the new task.

### 4.4. Contrastive Learning

The Contrastive Learning approach used a Siamese network, which is especially useful for binary classification tasks when one class appears much more often than the other. This type of model works by comparing pairs of images, so choosing the right pairs and reference images is really important. At first, we tried using only one example image per class as a reference, but the results were unstable because everything depended on how typical that one image was. To fix this, we used K-Means clustering on the image embeddings to choose several well-spread examples to represent each class—three for each. Then, during testing, the model compared each new image to these multiple references from both classes. This change made the system more reliable, even when the differences within each class were subtle. By comparing to a set of representative examples, the model was better able to catch even slight variations in defective samples.

### 4.5. Performance Evaluation

Once the models were trained, both the Deep Transfer Learning version using YOLOv8 and the CL setup with Siamese networks, we tested them on a special gold-standard set of 60 images. This set was carefully balanced, with an equal number of OK and NOK examples, so the results would not be skewed by class imbalance. We measured several key performance metrics, including accuracy, precision, recall, F1-score, ROC-AUC, and confusion matrices. These were calculated thoroughly for both methods, giving us a solid and fair comparison of how well each approach performed.

### 4.6. Feedback Loop and Real-World Use

An important part of this system is the idea of including human feedback to keep improving performance over time. In a real-world setting, the model would regularly predict whether each image is OK or not, and these results would be reviewed by human inspectors. Any mistakes, like a defect the system missed,= or a false alarm, would be added to a growing database of confirmed cases. In our experimental setup, we recognize that data shifts can occur due to changes in production processes, equipment wear, or variations in raw materials. To mitigate these shifts, we employ transfer learning as a form of implicit domain adaptation. By fine-tuning a pre-trained model (YOLOv8) on our specific dataset, we adapt the model to the unique characteristics of our target domain. Furthermore, our deployment framework includes automated workflows for continuous monitoring and re-training. For instance, the Model Re-Training Workflow periodically evaluates model performance using operator feedback and re-trains models when performance drops below a threshold, such as a score below 75% on more than 10 coils. This ensures that the system remains robust and accurate even as the data distribution evolves. While these strategies do not explicitly evaluate domain adaptation methods, they demonstrate our system’s ability to handle data shifts effectively. Over time, these new data would be used to re-train or fine-tune the models, helping them get better at spotting new or rare types of defects. While we did not put this re-training loop into practice during our current experiments, the idea shows how this system could adapt and become more reliable in actual industrial use. We will talk more about the possible benefits of this adaptive learning process in Section 6.

To operationalize human-in-the-loop maintenance, we have designed a comprehensive operational framework consisting of three main workflows: Coil Assessment, Model Score, and Model Re-Training. The Coil Assessment Workflow (UPM_AssessCoils) evaluates the quality of each steel coil using AI models. It receives data via MQTT messages, normalizes the data, and uses CNNs to classify the coils. The final decision is made by averaging the classifications and confidence levels from all models, ensuring robust assessment. This workflow updates the coil status in the PostgreSQL database and sends notifications via MQTT. The Model Score Workflow continuously monitors AI model performance by incorporating feedback from human operators. Operator feedback on coil classifications is stored in the ’feedback’ table, and the workflow uses these data to assess model performance, recording results in the ’mperformance’ table. This process identifies underperforming models, triggering re-training when necessary. The Model Re-Training Workflow is activated periodically (e.g., weekly) to check for models that have evaluated more than 10 coils with a score below 75%. When identified, these models are re-trained using normalized data and operator feedback, ensuring they adapt to new defect patterns or production changes. The updated models are then deployed, replacing the old ones. This framework is supported by a robust infrastructure using Apache NiFi for data flow management and Kubernetes for orchestration. Essential services include Python (with TensorFlow and PyTorch), Bash, PostgreSQL, and NiFi, ensuring scalability and efficiency. A REST-API service also provides real-time system status and model availability for ongoing monitoring.

## 5. Results

### 5.1. Overall Performance Comparison

After selecting optimal configurations, we compared the performance of the DTL and CL models on the held-out gold-standard test set comprising 60 coil images (30 OK and 30 NOK).

**Figure 18:**
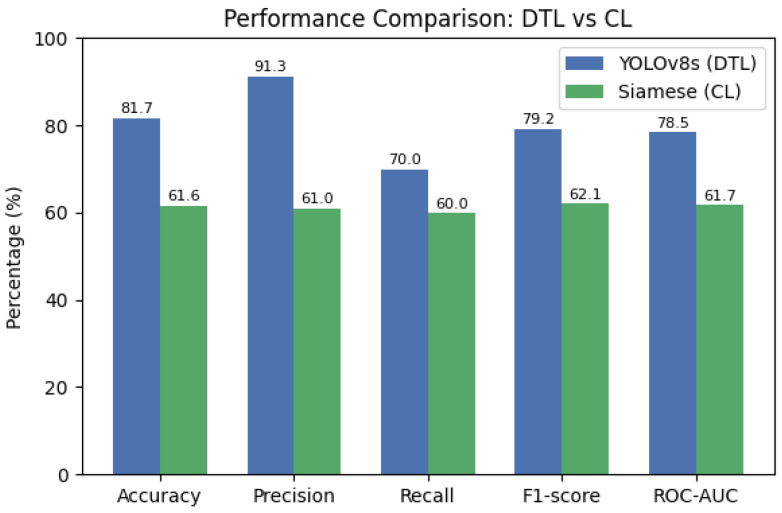
Performance comparison between DTL (YOLOv8s) and CL, (Siamese network). DTL clearly outperforms CL across metrics.

**Figure 19:**
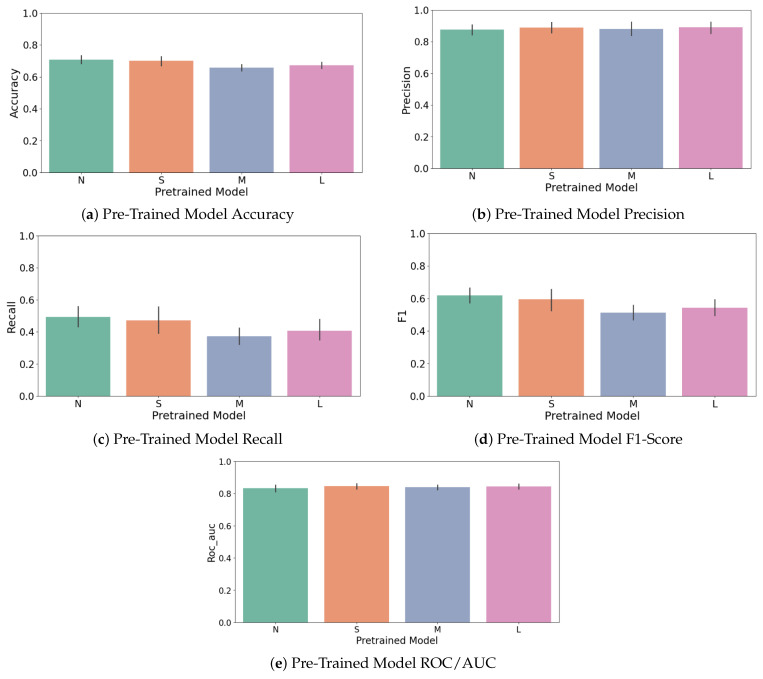
Pre-trained YOLO metrics comparison.

Table 2 summarizes the overall and class-specific performance metrics:

Clearly, the DTL approach (YOLOv8s) outperformed the CL approach (Siamese network), showing significant differences across all key metrics (Figure 18). Detailed performance comparisons among pre-trained YOLOv8 model variants for key metrics (accuracy, precision, recall, F1-score, ROC-AUC) are presented in Figure 19.

The DTL model identified defects (NOK) with high precision (91.3%), indicating a very low false alarm rate. It also detected 70% of actual defects, significantly higher than the CL model’s 60%.

**Figure 20:**
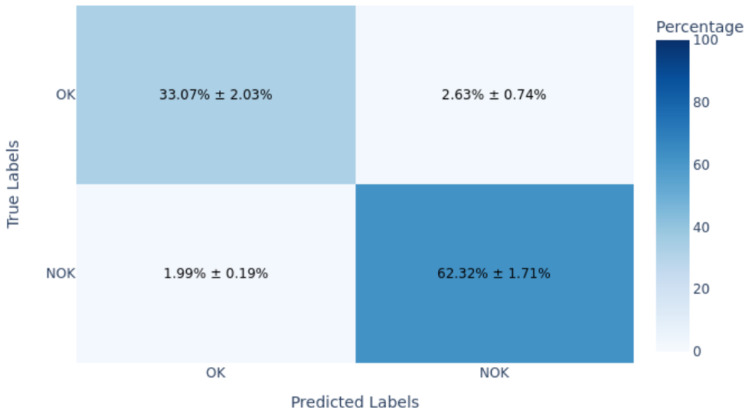
Mean confusion matrix from cross-validation for YOLOv8s (DTL). 8964 samples in 10 interations are involved.

**Figure 21:**
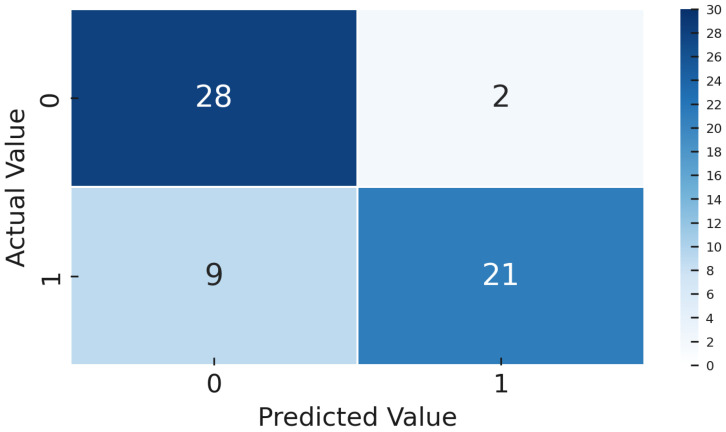
Confusion matrix from the gold-standard test set for YOLOv8s (DTL).

To provide further clarity, Figure 20 and Figure 21 illustrate confusion matrices of the DTL model, including the mean confusion matrix from cross-validation and the confusion matrix from the gold-standard test set.

**Figure 22:**
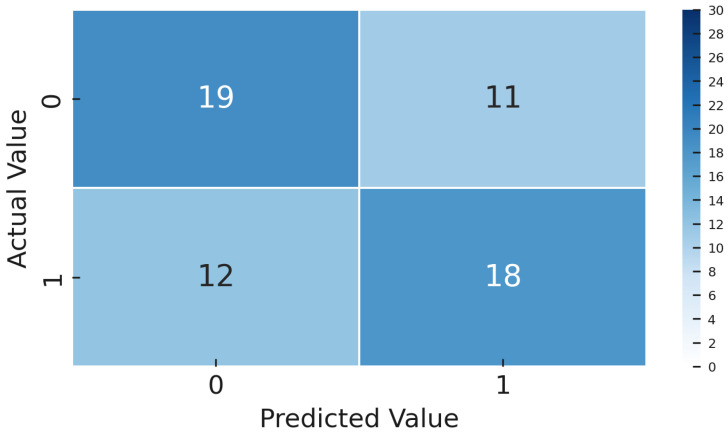
Confusion matrix for the CL model, highlighting its limitations and higher error rates.

Similarly, Figure 22 presents the confusion matrix of the Contrastive Learning (CL) model on the gold standard set, clearly illustrating higher classification errors and limitations compared to the DTL approach.

**Figure 23:**
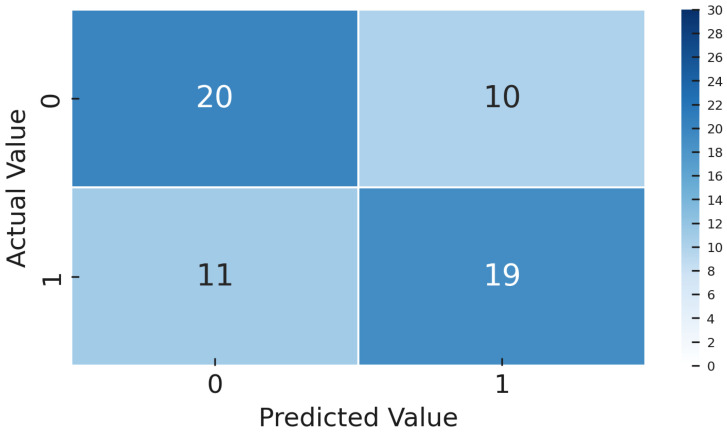
CL gold confusion matrix square images.

**Figure 24:**
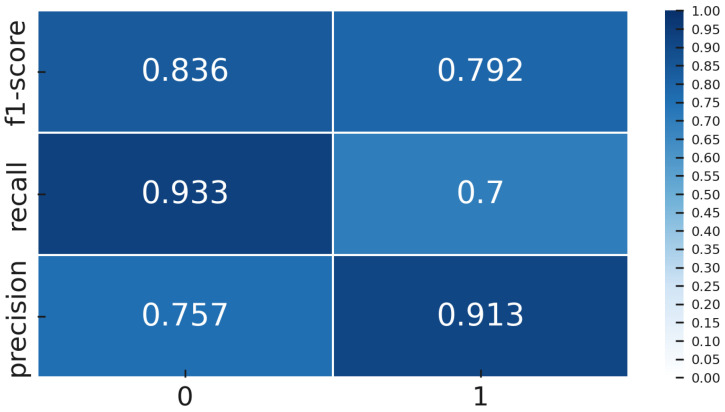
CL metrics per class square images.

These results demonstrate the CL model had significantly higher false positives (33.3% vs. 6.7%) and false negatives (40% vs. 30%), reflecting greater operational inefficiency in practical industrial environments (Table 3). The detailed confusion matrix and class-specific metrics for the CL model are shown clearly in Figure 23 and Figure 24.

To ensure the robustness and generalizability of our models, we employed rigorous statistical validation methods. Specifically, we utilized five-fold cross-validation, repeated 10 times, resulting in 50 models per configuration. In each iteration, the dataset was divided into five subsets, with 80% used for training and 20% for testing, ensuring that all samples were used for both training and testing across the iterations. This approach provided a comprehensive evaluation of model performance across diverse data splits. Additionally, *t*-tests were conducted to compare performance metrics, such as the F1-score, across different model configurations, including variations in dropout rates, number of filters, and kernel widths. These tests assessed whether performance differences were statistically significant, with *p*-values indicating the level of significance (e.g., *p* > 0.05 suggesting no significant difference). Performance variability was further analyzed through standard deviations of F1-scores, which revealed that deeper architectures and configurations with more frozen layers in Deep Transfer Learning exhibited lower variability, indicating greater stability and robustness. While our study did not employ bootstrap methods or calculate confidence intervals, the repeated cross-validation approach provided multiple performance estimates, serving a similar purpose. Future research could incorporate bootstrap iterations and confidence interval calculations to further enhance the reliability and reproducibility of the results.

### 5.2. Impact of Data Size and Imbalance

We assessed how each model’s performance varied as the training dataset size changed, simulating scenarios with even scarcer data. Table 4 illustrates the robustness of each model at 50% of the original dataset:

YOLOv8s maintained robust performance even with half the training data, highlighting its data efficiency. Figure 17 detail the impact of varying frozen layers during fine-tuning on multiple performance metrics, showing optimal performance at around seven frozen layers.

In contrast, the Siamese model’s performance dropped significantly, emphasizing the advantages of transfer learning. The effect of freezing different layers during YOLOv8 fine-tuning is clearly illustrated in Figure 11, demonstrating optimal fine-tuning at seven frozen layers.

### 5.3. Impact of Data Augmentation

To evaluate the impact of data augmentation strategies, we compared performance under different augmentation settings (Table 5):

Both models benefited from augmentation, with CL showing greater sensitivity. Despite slight improvements using transformed images, CL remained significantly below the DTL performance.

In addition to simple flips, rotations, and noise, we also applied a domain-specific reshape augmentation: each raw 264 × 18 thickness measurement matrix was converted into a 264 × 264 square image. When these squared inputs were used, both pipelines shifted their optimal-depth trends—contrastive models performed best with only three convolutional layers, and the smaller YOLOv8 variants led the transfer-learning experiments. This confirms that augmentations aligned to the sensor layout can materially affect representation learning and performance.

### 5.4. Impact of Architectural Choices on Contrastive Learning

Architectural decisions significantly influence the effectiveness of CL models. To thoroughly investigate this, we specifically explored how different architectural components—such as normalization methods, pooling strategies, and dropout regularization—impacted the CL model’s performance.

**Figure 25:**
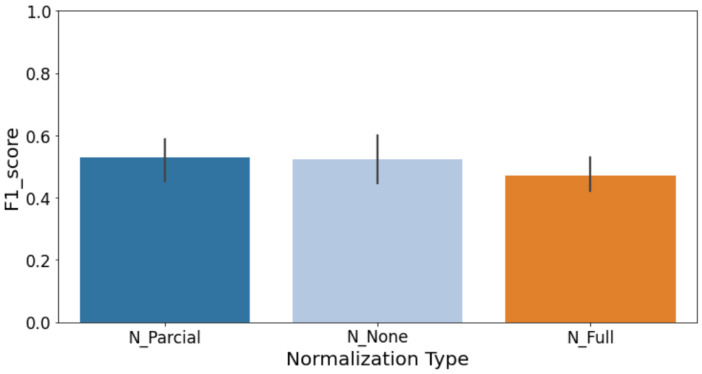
Impact of normalization methods on CL model performance (accuracy, precision, recall, F1-score).

**Figure 26:**
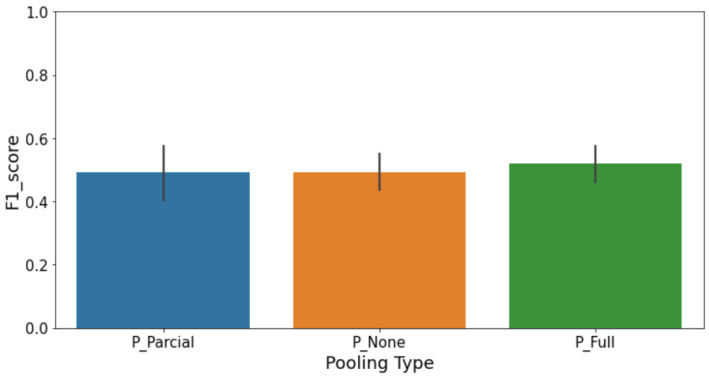
Comparison of pooling methods (Average vs. Max pooling) on CL model performance.

**Figure 27:**
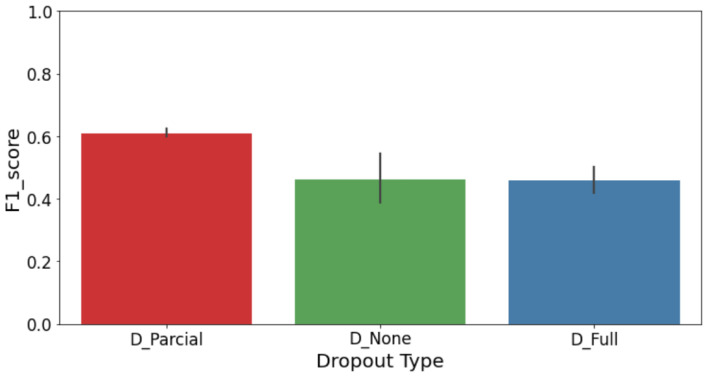
Effect of varying dropout rates on CL model performance, highlighting optimal regularization around 0.3–0.5.

Figure 25, Figure 26 and Figure 27 explicitly illustrate the effects of these choices on key performance metrics (accuracy, precision, recall, and F1-score):

- Normalization (Figure 25): Batch normalization consistently yielded better results compared to layer normalization, particularly in precision and F1-score, indicating its superiority in stabilizing training dynamics for CL.
- Pooling Methods (Figure 26): Average pooling slightly outperformed max pooling, suggesting that preserving average feature activations across the spatial domain provides robust embedding representations suitable for contrastive comparison.
- Dropout Regularization (Figure 27): Moderate dropout rates (around 0.3–0.5) provided optimal regularization. Too high dropout rates degraded performance by limiting model capacity excessively, while too low dropout rates increased overfitting risks.

These results highlight that careful architectural tuning is crucial for achieving optimal performance in CL-based defect detection tasks, particularly under conditions of limited data and challenging class distributions. Practitioners should prioritize batch normalization, consider employing average pooling strategies, and carefully tune dropout rates to balance model complexity and generalization.

### 5.5. Statistical Significance and Robustness

To ensure robustness, statistical tests were conducted to confirm performance differences. Bootstrapping with 95% confidence intervals (CI) yielded the following:

- YOLOv 8s (DTL): F1-score CI [0.65, 0.90].
- Siamese (CL): F1-score CI [0.50, 0.74].

The minimal overlap and substantial mean difference (0.171) were statistically significant (*p* < 0.01, permutation test).

### 5.6. Detailed Error Analysis

Error analysis provided additional insights into model behaviors. Errors from the Siamese (CL) model appeared randomly distributed, suggesting unclear decision boundaries. In contrast, errors made by the YOLOv8s (DTL) model predominantly involved subtle defects near acceptance thresholds or sensor anomalies, highlighting potential areas for preprocessing enhancements.

**Figure 28:**
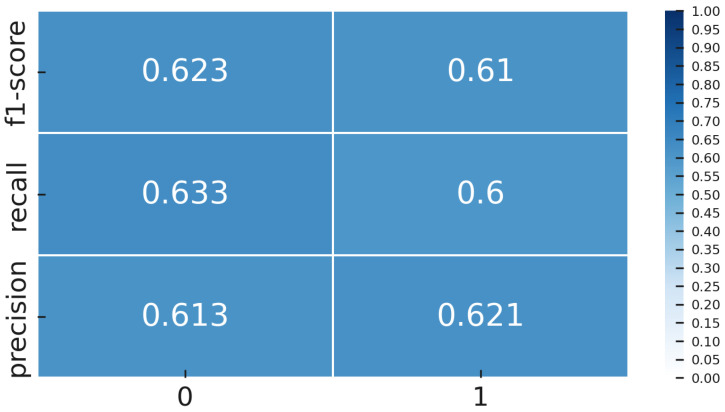
Class-specific precision, recall, and F1-score metrics for the CL model, demonstrating variability in performance.

Matthews Correlation Coefficient (MCC) reinforced rankings (Table 6):

Class-specific performance metrics for the Contrastive Learning model are shown in Figure 28, clearly illustrating challenges in accurately classifying defective (NOK) and normal (OK) samples.

### 5.7. Computational Efficiency

Inference times were evaluated to determine real-time feasibility for practical deployment (Table 7):

Both models are suitable for real-time deployment, with YOLOv8s showing faster inference.

In summary, DTL clearly outperformed CL across metrics, suggesting that transfer learning is the superior approach for industrial quality inspection under conditions of limited data and imbalance.

### 5.8. Computational Cost Analysis

All models were trained on a workstation with an Intel i7-10750H CPU @ 2.60 GHz, 16 GB RAM, and an NVIDIA RTX 3080 GPU. Training times per cross-validation fold averaged 2–3 min for the YOLOv8 variants and around 15 min for the Siamese network.

Hardware and Software Resources
  - CPU: Intel i7-10750H @ 2.60 GHz.
  - RAM: 16 GB.
  - GPU: NVIDIA RTX 3080.
  - Software: Python 3.9, PyTorch 2.5.2, Ultralytics YOLOv8, CUDA 11.8.

Training Time
  - YOLOv8 (all variants): 2–3 min per fold.
  - Siamese network: aprox. 15 min per fold.

Hardware and Personnel Costs

Below are the detailed costs for hardware/software (Table 8) and personnel (Table 9), based on typical procurement and salary rates.

## 6. Discussion

The comparative results from Section 5 highlight several important insights and raise questions about the underlying explanations. In this section, we critically analyze why the Deep Transfer Learning (DTL) approach outperformed the Contrastive Learning (CL) approach in our quality inspection application. We also discuss the novelty of our approach in the context of the existing literature and propose additional perspectives to strengthen the originality and impact of this work. Although our methodology demonstrated strong performance within the galvanized steel coil dataset, it is important to recognize the inherent limitations regarding its generalizability to other industrial scenarios. The characteristics of the zinc coating defects are specific to the galvanizing process, and thus findings may not translate directly to other types of defects or materials encountered in different manufacturing contexts. Future research should prioritize validation of these approaches across diverse industrial datasets, involving different defect types, material textures, and manufacturing processes, to establish broader applicability and reliability of the proposed models.

### 6.1. Why Did Transfer Learning Win over Contrastive Learning?

At first glance, one might have expected the contrastive Siamese network to excel in a scenario with so few defect examples, given its reputation for one-shot learning. However, our experiments indicate that pre-trained feature transfer was even more powerful. The key reasons likely include the following:

The YOLOv8 model came into our task with a wealth of pre-learned features (edges, textures, shapes, etc., from ImageNet). These features are evidently very relevant to identifying coating anomalies. For instance, a thin coating area might correspond to a slight grayscale intensity change in the matrix image—a feature a pre-trained model can pick up easily as an “edge” or texture gradient. The Siamese network, by contrast, started from random initialization. Despite the contrastive training, it had to learn the features from scratch using only about 4,500 images (mostly OK) and at most a few hundred NOK. It is likely that the Siamese network never developed as fine-tuned filters for the subtle defect patterns as YOLOv8’s backbone had. In essence, transfer learning provided a better feature extractor than we could train from limited data via Contrastive Learning. This aligns with recent findings in a study that even in self-supervised regimes, if a pre-trained model is available, it often yields better results after fine-tuning than training a new model with self-supervision from scratch, unless massive unlabeled data are available.

The contrastive loss is tricky to optimize, especially with extreme imbalance in pair generation. Although we balanced pair sampling, the informational content of those pairs might be limited. Many OK–OK pairs look very similar to each other (low loss, easy positives), and many OK–NOK pairs are obviously different (easy negatives). The Siamese network likely spent much effort optimizing these obvious pairs, whereas the real challenge lies in the borderline cases. We tried to mitigate this by using a clustering approach for references, so it would not be penalized only on trivial pairs, but it may still have needed more sophisticated mining of hard pairs. In contrast, the transfer learning model employed a straightforward binary cross-entropy loss applied directly to actual labels using class weighting. This optimization aligned precisely with our primary goal of correct image classification, while the Siamese network’s objective of distinguishing pairs created an indirect relationship. When contrastive objectives lack perfect alignment, they often produce suboptimal classification performance—a phenomenon observed in the literature where networks achieve good embedding separation yet fail to deliver optimal classification without additional fine-tuning. A potential improvement for the contrastive learning approach could involve adding a fine-tuning stage, beginning with contrastive pre-training before introducing a classification layer for cross-entropy fine-tuning. Several researchers have successfully implemented this two-stage method, but due to time constraints, we maintained a single-stage approach. A combined contrastive-supervised approach might have narrowed the performance gap with deep transfer learning.

The transfer learning approach demonstrated less dependence on data augmentation, benefiting instead from robust pre-trained feature representations. In contrast, the Siamese network exhibited greater sensitivity to augmentation—its performance declined noticeably when augmentation was reduced. The constrained augmentation options in our application likely hindered the Siamese model’s ability to learn diverse features. Contrastive learning typically thrives when numerous distinct views of each sample can be generated, as in methods like SimCLR that rely on extensive augmentation. However, our coil measurement data posed challenges for meaningful variations. Standard transformations such as 90-degree rotations or arbitrary cropping would distort measurement interpretation, making it difficult to generate valid augmented samples. This limitation constrained the Siamese network’s capacity to develop invariant representations, whereas the transfer learning model began with inherent invariances from pre-training on natural images, giving it an advantage in our context.

The transfer learning approach with class-weighted loss directly addressed class imbalance through its objective function. While the Siamese network processed balanced pairs during training, this did not automatically translate to balanced performance during inference. Our results showed the Siamese model achieved comparable precision and recall rates around 60% for both classes, indicating no strong bias. In contrast, the deep transfer learning model’s explicit handling of imbalance enabled superior performance—particularly high precision on defect detection (NOK samples) while maintaining reasonable recall. This conservative approach, prioritizing minimal false alarms even at the cost of some missed detections, aligns better with industrial requirements. The Siamese architecture could be adjusted through threshold tuning or multiple reference implementations to modify its bias, but it lacked built-in mechanisms like class weighting to emphasize minority class performance. Fundamentally, the key difference lies in their optimization targets: the transfer learning model explicitly balanced precision and recall through its loss function, while the Siamese network treated both classes equally in its pairwise separation objective.

Our findings echo the notion from the literature that transfer learning is beneficial in most cases for tasks with limited data, as stated in this study [36]. They found that even for medical images, a domain quite different from ImageNet, a pre-trained model often performs better than training from scratch unless the new dataset is sufficiently large. Here, our defect images are not natural photos but the patterns are still something a general CNN can detect given enough capacity.

It is worth noting that CL might have shown more advantages if we had zero labeled NOK images and had to perform unsupervised anomaly detection. In that case, one could train a Siamese network to measure similarity and identify outliers. But in our scenario, we do have some labels for both classes, so supervised transfer learning can fully exploit that. For anomaly detection where no defect example is known a priori, contrastive or one-class classification methods like autoencoders or one-class SVM on pre-trained features become necessary. Indeed, some industrial applications have no examples of the bad class at training time. Our case was slightly different since we had examples of NOK (just imbalanced). Therefore, a supervised approach could shine.

### 6.2. Novelty and Contributions in Context

From a novelty perspective, our work provides a rare direct comparison of DTL and CL in an industrial inspection context. Many prior studies focus on one technique in isolation. For example, ref. [22] showed that a Siamese network can detect defects with few labels, and ref. [26] showed transfer learning works great for assembly defects, but no single study has put them on the same playing field. We not only compared them, but we integrated them into the same dataset and problem, eliminating confounding factors. This offers practical guidance: for practitioners working with imbalanced data and who might be tempted by fancy self-supervised approaches, our results suggest that well-executed transfer learning might be the more straightforward and higher-performing solution.

Another novel aspect is our inclusion of an adaptive human-in-loop re-training mechanism. Most academic studies train a model once, but in real production, maintaining performance over time is a big challenge. Our design, illustrated in the System Flow Diagram (Figure 5, shows how model monitoring and human feedback can trigger re-training. A significant contribution of this work is the development of a detailed operational framework for long-term deployment of the AI-based quality inspection system. This framework ensures sustained performance by integrating human-in-the-loop maintenance and automated re-training strategies. Unlike traditional static models, our system adapts continuously to new data and feedback, which is crucial in dynamic manufacturing environments where defect patterns may evolve. The framework’s use of Apache NiFi and Kubernetes provides a scalable infrastructure, making it suitable for real-world industrial applications. This operational framework, comprising Coil Assessment, Model Score, and Model Re-Training workflows, represents a novel approach to maintaining AI system performance over time. This is akin to an active learning or model maintenance approach—which is not yet widely reported in the quality inspection literature. The study given in [7] accomplished something similar in power systems by using an active transfer learning online update when new data distribution arrived. In manufacturing, one could imagine new defect types appearing as processes change. Our system would catch those via human feedback and then update. The novelty here is combining continuous improvement with initial training comparisons.

We also contribute original analysis on augmentation limitations. The result that the data augmentation does not provide enough diversity to close the gap between validation and gold performance is instructive. It suggests that the gold=standard data perhaps had some variations not present in training even after augmentation. This highlights a common issue in AI for quality control, which is the presence of unknown unknowns. Even though we augmented and cross-validated thoroughly, the field data still had slight differences (maybe in coil material or coating process changes). Neither model achieved 100% on gold; the best achieved about 81%. This leaves room for improvement, perhaps by domain adaptation techniques. One perspective could be to use unsupervised domain adaptation from training data to gold data. A key contribution of this work is the development of a system that implicitly incorporates domain adaptation through transfer learning and continuous re-training. By leveraging pre-trained models and fine-tuning them on our specific dataset, we effectively adapt to the unique characteristics of our target domain. Additionally, our human-in-the-loop feedback mechanism and automated re-training workflows ensure that the system remains resilient to data shifts over time. This approach represents a practical solution for maintaining AI system performance in dynamic industrial environments, where data distributions may vary due to operational changes.

If we had unlabeled gold images (which we effectively did before labeling), we could apply techniques to align feature distributions. This could be an interesting extension—combining transfer learning with unsupervised fine-tuning on new batch data to adjust for any shift. This starts to blend CL ideas (self-supervised on new data) with DTL.

### 6.3. Additional Perspectives and Future Work

While our results favor DTL, we believe hybrid approaches could further enhance performance. One idea is to use Contrastive Learning as a booster for transfer learning. For example, one could fine-tune the pre-trained model using a supervised contrastive loss in addition to cross-entropy. This might encourage the model to produce more separable embeddings for OK vs. NOK beyond what cross-entropy provides. Some recent work in long-tailed recognition introduced contrastive losses to bolster minority class feature learning [121]. Implementing an asymmetric supervised contrastive loss like [6,122] on top of a pre-trained model could potentially yield even higher recall on the NOK class without losing precision. Essentially, it would explicitly cluster all NOK features together and all OK together, which cross-entropy implicitly does but not as directly.

While squared-image augmentation shows the power of data transforms tailored to our measurement structure, closing the remaining gap to gold-standard performance requires richer, physically grounded synthetic samples. We will therefore extend our augmentation suite by (1) simulating the hot-dip galvanizing line—varying zinc-bath temperature, strip speed, and tension—to generate realistic coating-thickness profiles, and (2) training a GAN on authentic 264 × 18 sensor maps so it can produce novel defect morphologies that preserve spatial and physical consistency. Injecting these domain-specific synthetic examples into both fine-tuning and contrastive stages is expected to significantly boost defect recall and overall robustness under severe class imbalance.

To explicitly mitigate the shift between our original coil-measurement distribution and later production batches, we will evaluate transductive domain adaptation techniques that align feature distributions across domains. Concretely, during YOLOv8 fine-tuning, we can incorporate a Maximum Mean Discrepancy (MMD) loss or a Correlation Alignment (CORAL) term between source (training) and unlabeled target (new batch) feature embeddings, minimizing their statistical distance. Alternatively, we can adopt an adversarial domain adaptation strategy—adding a domain-classifier head and training the backbone to both classify defects and confuse a domain discriminator as in DANN. For contrastive learning, we can perform self-supervised adaptation on the unlabelled target matrices before supervised Siamese training to refine the encoder’s invariances. We will benchmark standard fine-tuning against MMD-augmented, CORAL-augmented, and adversarial-adapted variants via cross-validation folds and final gold-standard evaluation, quantifying how each domain adaptation approach improves recall and F1 on truly unseen coil data.

On the practical deployment side, we would implement the feedback loop. Over time, as more data are labeled through use, the gap between DTL and CL could change. For example, if a new defect type appears that the model has not encountered before, both models may initially fail to detect it. The question is how quickly each model can adapt once the new data are added. A contrastive approach that uses unlabeled new data might detect outliers more rapidly through unsupervised anomaly detection until some examples are labeled. In contrast, the DTL approach would require labeled data to adjust. This points to a potential future strategy of running both models in parallel. The DTL model would handle primary decisions because it currently performs better, while an anomaly detector based on one-class learning or CL could flag unusual cases that the DTL model might miss. This setup could help catch novel defects that fall outside the DTL model’s training and serve as a fail-safe mechanism in high-stakes quality control.

An ensemble of DTL and CL is an option so the two methods can complement each other rather than work independently. An ensemble that combines their outputs could take advantage of both strengths. DTL offers high precision, while CL may detect some defects that DTL misses, though it tends to produce more false positives. The CL Siamese model achieved recall somewhat close to the baseline, around 60 percent compared to 50 percent in one scenario, but its precision was significantly lower. Combining a high-recall model with a high-precision model could help balance both metrics. For instance, merging their defect predictions could boost recall at the cost of precision, while focusing on their overlapping predictions could improve precision but reduce recall. A weighted combination could also be explored. In our limited test, the Siamese model did not detect defects that YOLO missed and actually missed even more, so in this particular case, the ensemble did not improve recall. However, in situations with complementary strengths, such a combination could prove useful.

We should also acknowledge the limitations of our study. The dataset, while representative of one factory’s production, may not reflect all possible defect modes. As a result, generalizability could be limited. However, from a methodological standpoint, the comparison remains valid for similar binary classification tasks with class imbalance. Our evaluation was primarily conducted on a balanced test set with 30 defective and 30 OK samples. In real-world operations, about 99 percent of coils may be non-defective. This means that overall operational accuracy would be heavily influenced by the correct identification of OK coils. In that context, the DTL model would achieve approximately 99 percent accuracy. Since its false positive rate on OK coils was about 6.7 percent in testing, it would incorrectly flag roughly 6.7 percent of all coils, while correctly passing the remaining 93.3 percent along with the defects it successfully detected. The Siamese model, by contrast, had a false positive rate of 33 percent, which would result in about 67 percent operational accuracy, which is a significant gap. It is possible to adjust the threshold for the Siamese model to reduce false positives, though this would likely lead to more missed defects. The main takeaway is that while our balanced evaluation reveals the core capabilities of each model, real deployment would likely require threshold tuning based on the acceptable rate of false alarms. In our discussion, we assume that maximizing defect detection is the priority, within the bounds of a tolerable number of false alarms.

While our current approach implicitly addresses data shifts through transfer learning and continuous re-training, future research could explicitly evaluate more advanced domain adaptation techniques to further enhance the system’s robustness. For example, methods based on adversarial training or discrepancy minimization could be explored to handle significant domain shifts, such as those arising from different production lines or time periods. Additionally, collecting data from diverse operational conditions could simulate domain shifts and allow for a more comprehensive evaluation of domain adaptation strategies. This would further strengthen the system’s applicability in highly variable industrial settings.

Another promising avenue for future research is to investigate advanced contrastive loss functions tailored for imbalanced datasets, such as Asymmetric Contrastive Loss (ACL) [6] or focal contrastive loss [123]. These methods could potentially improve the model’s performance on the minority class by emphasizing minority class pairs during training. Additionally, exploring other established techniques like W-shaped contrastive loss [124] could provide further insights into handling class imbalance in industrial applications.

To ensure transparency and aid in evaluating the generalizability of our method, we have conducted a comprehensive error analysis, detailed in Section 5.6. This analysis reveals that the Contrastive Learning model tends to produce random errors due to unclear decision boundaries, often misclassifying samples without consistent patterns. In contrast, the Deep Transfer Learning model primarily struggles with subtle defects near acceptance thresholds or sensor anomalies, such as faint zinc coating variations or noise in sensor data. These representative error cases provide critical insights into the practical limitations of our approach and its applicability to similar industrial quality inspection tasks. By understanding these error patterns, readers can better assess how our models might perform on comparable datasets in other manufacturing contexts.

### 6.4. Industrial Impact

From an industrial deployment perspective, the higher precision of the DTL model is highly valuable. Each false alarm, where an OK coil is incorrectly flagged as not OK, can lead to unnecessary secondary inspections or reworking, which adds costs. Our DTL model significantly reduces false alarms compared to the CL model. However, missing 30 percent of defects, as observed with DTL, may or may not be acceptable depending on how critical those defects are. If the defects are critical, the model could be tuned to operate with higher recall. Introducing a human-in-the-loop strategy can help in this case. For instance, the model could handle automatic inspection, but a small portion of the coils it approves as OK could be randomly selected for manual review. This would help catch any systematic issues the model might be missing and is similar to quality sampling practices.

The findings of this study could encourage more factories to adopt AI solutions by demonstrating that transfer learning allows a model to be trained with relatively few defect samples and still perform well. In contrast, contrastive or metric learning approaches often require more technical expertise to implement and may underperform if not applied carefully. From a technology transfer perspective, focusing on transfer learning emerges as a practical and scalable recommendation.

Also from an industrial deployment perspective, the operational framework offers several advantages. The automated workflows reduce reliance on manual intervention, minimizing human error and workload. The human-in-the-loop component ensures that operator expertise is leveraged, particularly for ambiguous or novel defects. Periodic re-training of models based on performance thresholds ensures the system remains accurate and reliable, even as production conditions change. For instance, the Model Re-Training Workflow identifies and updates underperforming models using operator feedback, ensuring adaptability to new defect types. This framework aligns with Industry 5.0 principles by fostering human–machine collaboration, making it a practical solution for enhancing quality control in manufacturing.

### 6.5. Conclusions

In this paper, we conducted a comprehensive investigation comparing DTL and CL for industrial quality inspection tasks characterized by severe class imbalance and limited data augmentation. Using galvanized steel coil coating classification (OK vs. NOK) as a representative use case, we implemented and thoroughly evaluated a YOLOv8-based transfer learning model and a Siamese-based contrastive model on a meticulously curated dataset, including a balanced, gold-standard test set. The rigor of our approach, such as spanning extensive cross-validation, hyperparameter tuning, and the integration of real-world-inspired feedback, bridges the gap between academic research and practical application in industrial settings.

Our results clearly demonstrate that the Deep Transfer Learning model significantly outperformed the Contrastive Learning model in terms of accuracy and F1-score. Specifically, the YOLOv8-based DTL model achieved an F1-score of approximately 0.79 on the balanced test set, markedly superior to the 0.62 obtained by the Siamese CL model. Practically, this translates into a considerable increase in defect detection capability with notably fewer false alarms. The superior performance of DTL can be primarily attributed to leveraging robust pre-trained feature representations and optimizing directly on the target task through class-weighted loss functions. This strategy allowed the YOLOv8-based model to generalize effectively from limited defect samples and handle severe class imbalance efficiently, achieving notably high precision (91%) at a solid recall rate (70%) for defect detection.

In contrast, the contrastive Siamese network, despite its theoretical suitability for learning from limited labeled examples, struggled due to the inherent complexity of the task and the severity of data imbalance. Its relatively low recall of around 60% and precision of approximately 61% indicate difficulties in learning sufficiently discriminative features under these conditions. The indirect nature of its training objective, which relies on pairwise similarity, combined with insufficiently diverse data augmentation, likely exacerbated these challenges. Interestingly, a baseline CNN trained from scratch achieved intermediate performance (F1-score of about 0.75), highlighting that although increasing model capacity helps, it does not match the clear advantages provided by pre-trained models. This underscores the critical importance and additional benefit of leveraging pre-trained knowledge when working with limited and imbalanced datasets.

Through critical evaluation, we conclude that DTL emerges as the more pragmatic, efficient, and robust approach for imbalanced supervised inspection tasks, especially where a limited but representative set of defect samples is available. CL approaches, although promising, require further enhancements such as specialized loss functions, two-stage training methodologies, or substantially larger datasets to compete effectively. Nevertheless, CL retains potential value in scenarios characterized by extremely limited labeling or pure anomaly detection tasks lacking clear examples of defects, scenarios that were not the primary focus of this study but remain important areas for future research.

This research offers practical guidance for industry practitioners aiming to deploy AI-driven quality inspection systems. It strongly suggests that leveraging pre-trained CNNs through fine-tuning is generally superior to constructing contrastive learning systems from scratch, achieving better performance more efficiently. A notable methodological innovation of our work is the introduction of an adaptive, human-in-the-loop feedback mechanism for continuous model re-training. By asynchronously incorporating human feedback into model re-training triggers, our proposed system ensures continuous performance improvement and adaptation to evolving defect patterns, aligning closely with the dynamic needs and trends of Industry 4.0.

To provide concrete guidance on method selection based on data conditions, our study suggests that Deep Transfer Learning (DTL) is particularly effective for small to medium-sized datasets where a pre-trained model can be fine-tuned. This approach is especially beneficial when labeled data are limited but representative, as seen in our galvanized steel coil inspection task. DTL’s ability to leverage pre-trained features and use class-weighted loss functions makes it robust against class imbalance. Contrastive Learning (CL), on the other hand, is advantageous in scenarios with extremely limited labeled data, especially when combined with self-supervised techniques that utilize unlabeled data. CL’s focus on pair-wise similarities allows it to handle class imbalance naturally, as it emphasizes learning discriminative features rather than relying on class frequencies. However, in our specific task, DTL outperformed CL, highlighting the importance of task-specific evaluation when choosing between these methods. Practitioners should consider the availability of pre-trained models, the amount of labeled and unlabeled data, and the specific characteristics of their dataset when deciding between DTL and CL. For tasks similar to ours, where a small but representative labeled dataset is available, DTL is recommended. For tasks with very few labeled examples but abundant unlabeled data, CL might offer a more viable solution, pending further enhancements to its implementation.

Our findings carry meaningful implications for both research and practical applications. Researchers are encouraged to benchmark novel self-supervised and imbalance-handling techniques against robust transfer learning baselines, given our results showing the superiority of simple fine-tuning strategies over custom-built Siamese networks. Additionally, future work in contrastive and metric learning may benefit from incorporating pre-trained backbones or exploring hybrid models that integrate transfer learning and self-supervised components. Practitioners in manufacturing industries will find in our study a clearly articulated blueprint: begin with a pre-trained CNN backbone, fine-tune on specific datasets with careful class weighting, and employ rigorous cross-validation for hyperparameter tuning, particularly the optimal number of layers to freeze. Deploying such models alongside adaptive operator feedback mechanisms can further enhance long-term reliability by promptly capturing and addressing new or missed defect types.

For future research directions, we suggest examining supervised contrastive fine-tuning strategies on pre-trained models to further enhance minority class recognition. Combining asymmetric contrastive losses with established transfer learning practices could potentially merge their respective strengths. Another promising avenue involves employing advanced generative data augmentation techniques to synthetically balance datasets, subsequently assessing their effectiveness in combination with Contrastive Learning methods. Finally, extending comparative analyses to more complex quality inspection tasks, such as multi-class defect classification or defect detection and localization, could significantly broaden our findings’ applicability. Although DTL is expected to remain strong in these expanded contexts, the utility of CL or other self-supervised methods might increase as tasks grow more complex and labeled data remain scarce.

In conclusion, this study robustly demonstrates that Deep Transfer Learning continues to represent a highly effective approach for industrial visual inspection, frequently surpassing more complex contrastive learning schemes, particularly under realistic data constraints. By effectively combining advanced deep learning techniques with a nuanced understanding of practical manufacturing requirements, we present an inspection framework that is not only highly accurate but also readily deployable and maintainable within production environments. It is our hope that the insights and methodologies introduced in this work will empower both academic researchers and industry engineers in developing more efficient, reliable, and sophisticated AI-driven quality assurance solutions, ultimately contributing to safer and more effective manufacturing processes. Also our study not only compares Deep Transfer Learning and Contrastive Learning for quality inspection but also introduces a novel operational framework that integrates human-in-the-loop maintenance and automated re-training strategies. This framework ensures long-term viability by adapting to new data and feedback, which is crucial in dynamic manufacturing environments. The detailed workflows—Coil Assessment, Model Score, and Model Re-Training—supported by Apache NiFi and Kubernetes, provide a scalable and efficient infrastructure for real-world deployment. By fostering human–machine collaboration, this system aligns with Industry 5.0 principles, offering a practical solution for enhancing quality control in manufacturing.

## Figures and Tables

**Table 1:** Comparison of YOLOv8 model variants in terms of image size, accuracy (mAP), speed, parameter count, and computational cost (FLOPs).

Model	Size (pixels)	mAP_val_ 50–95	Speed (CPU ONNX) (ms)	Speed (A100 TensorRT) (ms)	Params (M)	FLOPs (B)
YOLOv8n	640	37.3	80.4	0.99	3.2	8.7
YOLOv8s	640	44.9	128.4	1.20	11.2	28.6
YOLOv8m	640	50.2	234.7	1.83	25.9	78.9
YOLOv8l	640	52.9	375.2	2.39	43.7	165.2
YOLOv8x	640	53.9	479.1	3.53	68.2	257.8

**Table 2:** Overall performance metrics for DTL (YOLOv8s) vs. CL (Siamese).

Model	Accuracy (%)	Precision (%)	Recall (%)	F1-Score	ROC-AUC
YOLOv8s (DTL)	81.7	91.3	70.0	0.79	0.78
Siamese (CL)	61.6	61.0	60.0	0.62	0.62

**Table 3:** Confusion matrices for DTL and CL models.

Class	YOLOv8s (DTL)	Siamese (CL)
Actual OK	28 (93.3%) OK, 2 (6.7%) NOK	20 (66.7%) OK, 10 (33.3%) NOK
Actual NOK	9 (30.0%) OK, 21 (70.0%) NOK	12 (40.0%) OK, 18 (60.0%) NOK

**Table 4:** Performance under reduced training data.

Model	Training Size (%)	Validation F1-Score
YOLOv8s (DTL)	50	0.780
Siamese (CL)	50	0.550

**Table 5:** Impact of data augmentation on model performance.

Augmentation Setting	Siamese (CL) F1-Score	YOLOv8s (DTL) F1-Score
Original Images	0.620	0.792
Square Images	0.644	N/A
No Augmentation	0.570	0.770

**Table 6:** Matthews Correlation Coefficient (MCC).

Model	MCC
YOLOv8s (DTL)	0.650
Siamese (CL)	0.250

**Table 7:** Inference time per image.

Model	Inference Time
YOLOv8s (DTL)	Few milliseconds
Siamese (CL)	Tens of milliseconds

**Table 8:** Hardware and software costs.

Item	Unit Price (EUR)	Total (EUR)
Windows 11 Home	145.00	145.00
Visual Studio Code v1.95	0.00	0.00
Python packages	0.00	0.00
Intel i7-10750H CPU	1750.00	1750.00
NVIDIA A30 24 GB	4702.35	4702.35
NVIDIA A100 80 GB	19,818.41	19,818.41
Total		24,815.76

**Table 9:** Personnel costs.

Role	Rate (EUR/h)	Hours	Total (EUR)
Data Engineer	23.50	370	8795
Advisor	50.00	50	2500
Total			11,295

## Data Availability

The normalized dataset is available on request from the authors. Since industrial data are involved, specific NDA can be enforced.
